# Reanalysis of the DEMS Nested Case-Control Study of Lung Cancer and Diesel Exhaust: Suitability for Quantitative Risk Assessment

**DOI:** 10.1111/risa.12371

**Published:** 2015-04-10

**Authors:** Kenny S Crump, Cynthia Van Landingham, Suresh H Moolgavkar, Roger McClellan

**Affiliations:** 1 Ruston, LA, USA; 2 ENVIRON International Corp1900 North 18th Street Suite 804, Monroe, LA, USA; 3 Exponent, IncBellevue, WA, and Menlo Park, CA, USA; 4 Albuquerque, NM, USA

**Keywords:** Diesel engine exhaust, lung cancer, nonmetal mines, risk assessment

## Abstract

The International Agency for Research on Cancer (IARC) in 2012 upgraded its hazard characterization of diesel engine exhaust (DEE) to “carcinogenic to humans.” The Diesel Exhaust in Miners Study (DEMS) cohort and nested case-control studies of lung cancer mortality in eight U.S. nonmetal mines were influential in IARC’s determination. We conducted a reanalysis of the DEMS case-control data to evaluate its suitability for quantitative risk assessment (QRA). Our reanalysis used conditional logistic regression and adjusted for cigarette smoking in a manner similar to the original DEMS analysis. However, we included additional estimates of DEE exposure and adjustment for radon exposure. In addition to applying three DEE exposure estimates developed by DEMS, we applied six alternative estimates. Without adjusting for radon, our results were similar to those in the original DEMS analysis: all but one of the nine DEE exposure estimates showed evidence of an association between DEE exposure and lung cancer mortality, with trend slopes differing only by about a factor of two. When exposure to radon was adjusted, the evidence for a DEE effect was greatly diminished, but was still present in some analyses that utilized the three original DEMS DEE exposure estimates. A DEE effect was not observed when the six alternative DEE exposure estimates were utilized and radon was adjusted. No consistent evidence of a DEE effect was found among miners who worked only underground. This article highlights some issues that should be addressed in any use of the DEMS data in developing a QRA for DEE.

## 1.INTRODUCTION

There has been a long-standing interest in determining whether chronic exposure to diesel exhaust poses a carcinogenic hazard and, if so, the extent of the hazard.^1^–^8^ This interest relates primarily to the presence in diesel engine exhaust (DEE) of respirable elemental carbon (REC) particles with associated organic compounds. In 1987, the International Agency for Research on Cancer (IARC) concluded, based in part on “limited human evidence,” that DEE was a “probable human carcinogen.”^3^

Following the IARC hazard characterization decision in 1989, two courses of action ensued: the conduct of more detailed risk assessments, and the conduct of additional epidemiological studies. In the United States, the National Institutes of Occupational Safety and Health (NIOSH), the Mine Safety and Health Administration (MSHA), the Environmental Protection Agency (EPA), and the California Air Resources Board (CARB) conducted risk assessments. All four agencies concurred with IARC’s hazard characterization for DEE. However, NIOSH, MSHA, and EPA concluded that the human evidence was not sufficient for developing quantitative risk assessments (QRAs) for DEE. Taking a contrary position, CARB proceeded to develop quantitative estimates of the lung cancer risk for DEE primarily based on the analysis of Dawson and Alexeeff,^9^ which used data from a cohort study of railroad workers. The World Health Organization^10^ concluded the human evidence was not sufficient for QRA and, alternatively, developed an estimate of potency for DEE to cause lung cancer using data from studies in rats exposed to DEE.

The IARC determination also served to stimulate additional epidemiological research. One of the major studies undertaken was the Diesel Exhaust in Miners Study (DEMS), jointly funded and conducted by NIOSH and the National Cancer Institute (NCI). The DEMS research involved three phases: (1) extensive efforts to develop estimates of the exposure of workers to REC, the indicator selected as a measure of DEE exposure; (2) a cohort analysis; and (3) a nested case-control study that considered smoking. The efforts to develop exposure estimates to REC are described in five papers^11^–^15^ by the DEMS investigators. A sixth paper by Crump and Van Landingham^16^ evaluates the DEMS exposure estimates and offers alternative exposure estimates for REC. The cohort analyses^17^ conducted by the DEMS investigators found lung cancer mortality to be positively associated with exposure to REC. The nested case-control study^18^,^19^ considered cigarette smoking as well as REC exposure, and concluded that in addition to the expected strong effect of cigarette smoking on lung cancer hazard, there was also a positive association between REC and lung cancer mortality.

Based on the availability of new data on the carcinogenic hazards of exposure to DEE, IARC in 2012 updated its earlier review of DEE. The results of the DEMS cohort study^17^ and case-control study^18^,^19^ were influential in IARC’s conclusion that the human evidence of carcinogenicity was now “sufficient” and that DEE was a “human carcinogen.” The 2012 IARC^20^ determination has renewed interest in the development of quantitative estimates of lung cancer risk for DEE, with the DEMS results being perceived as a leading candidate for use in developing a QRA for DEE. In that regard, the EPA and various engine manufacturers requested that the Health Effects Institute (HEI), a nonprofit entity jointly funded by government and industry, convene a special panel of experts to review all of the available epidemiological literature, including particularly the DEMS study, and to offer an opinion on its suitability for use in QRA. Interest in developing a QRA for DEE exposure and the anticipated use of the DEMS results motivated us to critically evaluate the original DEMS data and make our results available to the HEI Panel.

The data requirements for a QRA are more stringent than for studies that establish only the existence of a relationship between exposure and risk, i.e., existence of a hazard, without attempting to develop a quantitative relationship. Whereas a relatively crude surrogate for exposure may be sufficient for establishing that exposure is correlated with disease, in a QRA, accuracy in quantitative estimates of exposures is as important as accuracy in the data on disease outcomes for estimating the potency of the agent for causing disease. Here, we present results replicating the original published analyses of the case-control study^18^ and providing additional analysis of the DEMS case-control data using several alternative estimates of DEE exposures not considered previously, with the goal of assisting in evaluating DEMS possible use in a QRA for DEE. Our alternative estimates were developed without any knowledge of how they would affect the analysis of the DEMS epidemiological data.

Our extended analyses differed from those of Silverman *et al*.^18^ in four ways: (1) we used alternative REC exposure estimates; (2) we made adjustments for radon (a well-known human lung carcinogen); (3) we made use of two trend tests, including one that utilized individually estimated REC exposures; and (4) we conducted additional analyses that considered individuals who worked only underground, and who were presumed to be the workers most heavily and consistently exposed to DEE. A separate paper^21^ reports the results of a reanalysis of the DEMS cohort data.^17^

Our analyses utilized the DEMS data made available to us by NIOSH and NCI, with access to the data controlled by the National Center for Health Statistics (NCHS), as described in the Supporting Information. We appreciate the cooperation of the original DEMS investigators and NCHS in making the DEMS data available for our work. Whenever a complex study such as DEMS is to be used to inform or establish public policy, it is prudent to allow others to conduct additional evaluations to determine the robustness of the original conclusions.

## 2.OVERVIEW OF DEMS

A detailed description of the DEMS research is provided in eight papers authored by NIOSH and NCI investigators.^11^–^15^,^17^–^19^ The DEMS study included 12,315 workers who worked as miners or in associated surface operations for at least one year in one of eight U.S. nonmetal mines, including one limestone mine (Missouri, labeled A), three potash mines (New Mexico, labeled B, D, and J), one salt mine (Ohio, labeled E), and three trona mines (Wyoming, labeled G, H, and I). The vital status of the workers was followed through December 31, 1997. The eight nonmetal mines were selected for study, in part, because the ores being mined were considered to be noncarcinogenic. Seven mines (trona, potash, and salt) were underground mines, with ore transported to central underground locations, typically by conveyer, and lifted to the surface. Those mines made substantial use of electric-powered equipment supplemented by diesel-powered equipment. They also made substantial use of mechanical ventilation. The limestone mine was different, with ore mined on a single plane and hauled laterally to the surface with large diesel-powered haul-ore units. That mine depended primarily on natural ventilation.

The DEMS study was larger than most other cohort studies of DEE and lung cancer (200+ lung cancer deaths identified), and estimates of DEE exposures were also larger than in earlier studies. The number of workers by mine type and work location is provided in Table SI of the Supporting Information. The DEMS analyses considered all the workers together or divided them into two subgroups, surface-only workers and ever-underground workers. The latter group included individuals who worked both underground and on the surface at different times.

## 3.DEE EXPOSURE ESTIMATES USED IN THE DEMS CASE-CONTROL ANALYSIS

The DEE estimates used in both the cohort study^17^ and in the case-control study^18^,^19^ are described in five publications.^11^–^15^ We provide a brief description here of how those estimates were defined. DEE is a complex mixture, and DEMS used REC as a surrogate for DEE. However, the only measurements of REC came from monitoring surveys of seven of the eight mines conducted by DEMS in 1998–2001 after follow up of the miners was completed, plus a small number of personal samples from a feasibility study conducted by DEMS in one of the mines in 1994 (see Table SII of the Supporting Information for additional information.) In the absence of historical measurements of REC, it was necessary to use data on other contaminants to estimate REC exposures. Information on airborne contaminants in the mines that could possibly be used as a surrogate for REC exposures included historical measurements of a number of gaseous contaminants (CO, CO_2_, NO, and NO_2_), gathered from surveys that had been conducted in the mines.^11^ In addition, information on the diesel equipment (type, use, and horsepower [HP]) and ventilation rates by year in each mine were developed by the DEMS team and, secondarily, made available to us. The Adj_HP (HP adjusted for the percent of time each piece of diesel equipment was estimated to have been operating) and ventilation rates (CFM, in cubic feet per minute) for each mine over the course of the DEMS study are shown in Fig. S1 in the Supporting Information. Those graphs show marked differences in both Adj_HP and ventilation in the different mines and, especially, differences among operations mining different types of ore, i.e., limestone, potash, salt, and trona. The relatively high Adj_HP in the limestone mine relates to the use of high HP units to haul the ore. As noted earlier, that mine depended primarily on natural ventilation. The relatively high ventilation in the trona mines relates to the need for ventilation to keep methane levels low to avoid its explosive hazard.

To estimate REC levels for years prior to 1998–2001, when REC measurements were not available, DEMS used CO as a surrogate for REC. However, CO measurements were available only beginning in 1976, whereas diesel equipment began being used in the 1960s in three mines, in the 1950s in four mines, and in 1947 in one mine. To estimate CO levels throughout the period when diesel equipment was used, DEMS used a mine-specific statistical model that regressed the natural logarithm (Ln) of CO on, among other determinants, yearly estimates of Ln of Adj_HP/CFM, and Ln of Adj_HP_1990+_ (adjusted HP using only diesel equipment installed in or after 1990).^14^ The limestone mine did not use mechanical ventilation, and in the regression equation for this mine, Adj_HP was used in place of the ratio.

To determine the relationship between REC and CO, DEMS conducted a regression analysis of CO and REC data from 1998 to 2001, obtaining a relationship of CO ∼ REC^β^ with a best estimate of β = 0.58.^11^ A large percentage of CO samples (47%), however, were below the detection limit. Values for those samples were imputed (assigned) using a statistical procedure, and those imputed values were used in the CO regression model.^14^

The DEMS investigators developed four REC estimates.^14^ In the REC estimate used in the case-control analysis,^18^ essentially mine-, department-, and job- specific estimates of REC derived from 1998 to 2001 DEMS survey were multiplied by the ratio, raised to the power β, of the model-estimated CO level for a given year to the corresponding CO estimate for the year the DEMS survey was conducted. However, β = 1 was used instead of the best estimate of β = 0.58. To estimate above-ground exposures, mine-, job-, and department-specific REC estimates obtained from the DEMS survey conducted in 1998–2001 were assumed to hold for all years diesel equipment was operated at a mine.

Mine J was closed in 1993 and consequently was not included in the 1998–2001 survey. REC estimates for that mine were based on the 1998–2001 REC data for mine B, which, like mine J, was a potash mine, and applying the determinants for mine J to the CO regression model for mine B.

In our analyses, we utilized three of the four DEE exposure estimates developed by DEMS:^14^

1. **DEMS_REC1**—estimates of REC developed by DEMS with β = 1, and used in the case-control study.^18^
2. **DEMS_REC2**—based on five-year average CO values for years after 1976, and the ratio of Adj_HP over ventilation (CFM) before 1976.
3. **DEMS_REC3**—same as DEMS_REC1 except assuming that β = 0.58 instead of β = 1.

DEMS_REC1 was the REC estimate used by Silverman *et al*. in the case-control study.^18^ DEMS_REC2 and DEMS_REC3 were applied in the addendum published subsequently by Silverman *et al*.^19^ We did not investigate the third alternative DEMS estimate^14^ because it is very similar to DEMS_REC1, differing only by summarizing the 1998–2001 REC data using medians rather than averages.

## 4.ALTERNATIVE EXPOSURE ESTIMATES

In a previous paper,^16^ we reviewed the REC exposure estimates developed in the DEMS study and developed an alternative estimate of exposures using assumptions similar to, but different from, those used by DEMS, but that seemed to us to be at least as credible as those used by DEMS. Here, in addition to applying that REC estimate to the case-control data, we explored the effect of various assumptions used to develop the DEMS REC estimates by making a number of alternative REC estimates based on modifying various steps in the DEMS estimation procedure, and using those alternative estimates to analyze the case-control data. All of these alternative REC estimates were developed independently of any consideration as to how they might influence the conclusions from the DEMS study.

After reviewing the information available on other gaseous contaminants in the mines (CO_2_, NO, and NO_2_) (see Supporting Information Table SII), we agreed with the DEMS investigators that, although (as discussed later) there were problems associated with using CO as a surrogate for REC, the shortcomings in the data available for the other gaseous contaminants were even greater, and so we developed several alternative estimates of REC exposures based on CO. Using the data from 1998 to 2001 DEMS survey, Crump and Van Landingham^16^ obtained a best estimate of REC ∼ CO^β^ where β = 0.3, as opposed to the value β = 0.58 obtained by Stewart *et al*.,^11^ or the value of β = 1.0 assumed in developing the REC estimates used by Silverman *et al*.^18^ Crump and Van Landingham^16^ also assigned alternative values to CO nondetect samples by using a statistical approach that was similar to that used by Vermeulen *et al*.,^14^ but that gave longer tails to the CO distributions (Fig. S2 of Supporting Information), applied those imputed CO values in the same CO regression model that was used by Vermeulen *et al*.,^14^ and used the results in making alternative REC estimates. In view of the substantial uncertainty in the relationship between CO and REC, we also elected to develop another alternative REC estimate based only on Adj_HP and mine ventilation data (CFM) for each mine (independent of the CO data).

Based on the work of Crump and Van Landingham,^16^ we defined the following alternative estimates of REC exposures and applied them in analysis of the case-control data. Additional explanation and description are provided in the Supporting Information.

1. **REC1**—REC estimates developed in Crump and Van Landingham^16^ (same as DEMS_REC1 except using independent estimates of 1998–2001 mine-, department-, and job-specific REC values, independently imputed CO values, and β = 0.3).
2. **REC2**—DEMS included a variable in their CO regression called “High Period” for mine H (only), stating “the parameter estimate for the observed high period in mine H was not included in the prediction model because the high concentrations could not be explained and only occurred for 2 years.”^14^ (Actually, because the REC estimates are based on a ratio of CO estimates, it would not have changed the REC estimates if the parameter estimate had been retained in the prediction model.) However, the “High Period” variable was retained in the regression model and therefore affected the estimates of other parameters. Herein, we investigate the effect of the “High Period” variable by defining REC2 in the same way as REC1, except not including “High Period” in the CO regression model for mine H.
3. **REC3**—Vermeulen *et al*.^14^ included a term in their regression CO model for six of the eight mines for HP installed after 1990, stating that the period after 1990 “corresponded to the introduction of cleaner direct injection engines and cleaner fuels.”^22^ However, there is no reference in the Haney and Saseen paper^22^ to the time frame of those improvements. Also, MSHA did not require EPA emission standards to be met in diesel equipment in mines until 2001, and then only for particulate matter, not CO.^23^ Knowledgeable persons with whom we consulted about this issue opined that 1990 was too early for the effects of those improvements in diesel technology to have influenced DEE emissions in the mines. Herein, we explore the effect of including this term in the model by defining REC3 in the same way as REC2, except using CO regression models that do not include the term for HP installed after 1990. This modification does not affect the exposure estimates for the two mines for which this term was not included by Vermeulen *et al*., and consequently allows the eight mines to be treated in a more uniform manner. β = 1 was used in this REC estimate.
4. **REC4**—same as REC3, except β = 0.3.
5. **REC5**—using three-year averages of CO samples for post-1975 CO estimates (see Supporting Information for additional details), β = 0.3.
6. **REC6**—estimating the REC in any given year relative to the measured level in 1998–2001 DEMS survey by the ratio of Adj_HP/CFM for the given year divided by the corresponding ratio obtained from the DEMS survey (independently of the CO data).

**Figure 1:**
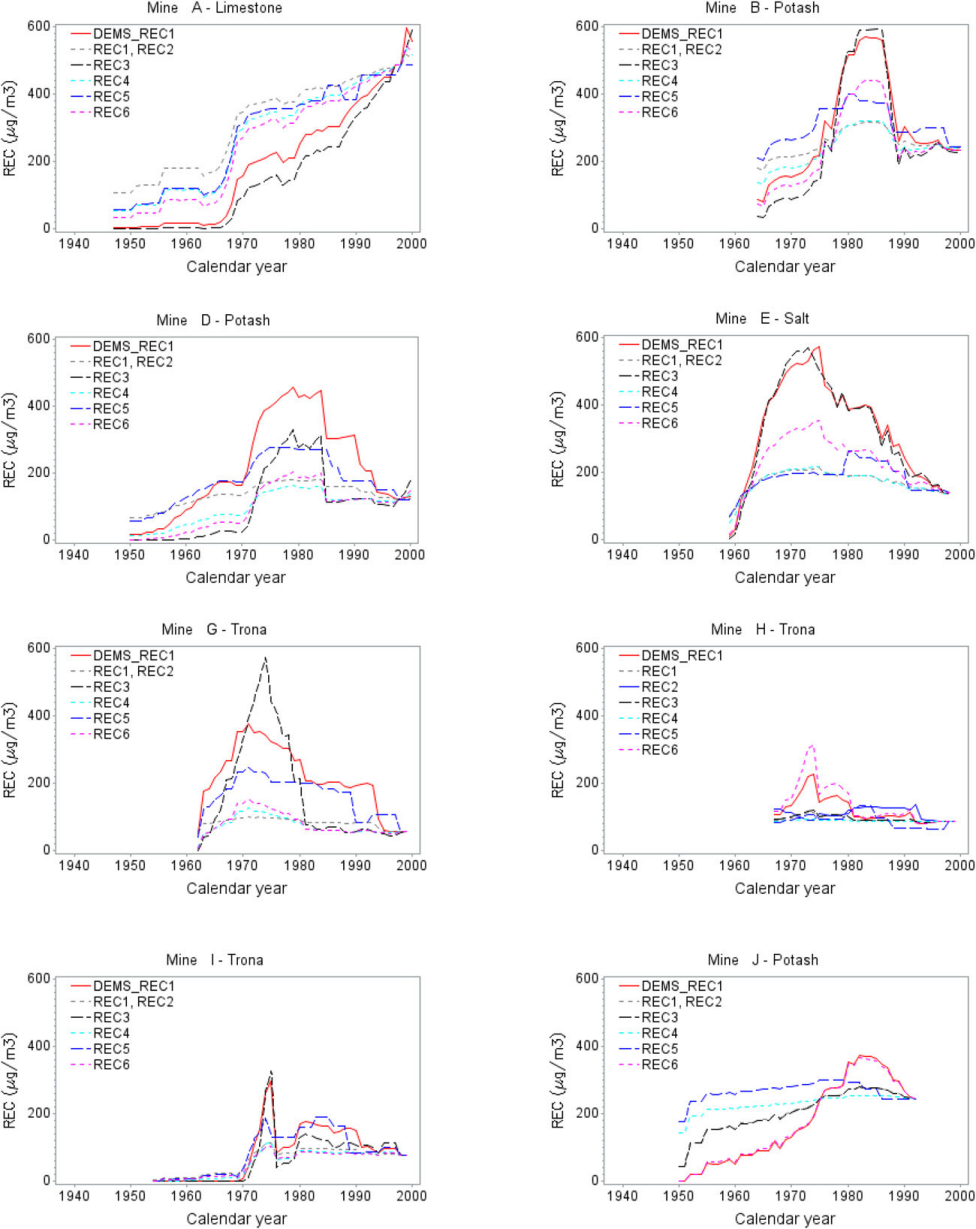
Mine-specific REC historical predictions for the underground job, mine operator. Mine A had no mine operator job, and REC estimates for the underground job, loader operator, are shown.

Fig.1 shows plots of the six alternative REC estimates for each mine, compared with the estimate (DEMS_REC1) used originally in the case-control analysis.^18^ These estimates appear different enough that they could have different implications for the association of REC with lung cancer. We explored this issue by applying each of the REC estimates in analyzing the case-control data. We note that many of the REC estimates for a mine have similar shapes, if different amplitudes. In particular, many of the estimates have a shape similar to that of REC6, which, unlike the other REC estimates, does not involve CO data at all. This similarity is because all that the CO data contribute to a REC estimate are two regression coefficients from the CO model, one of which affects only the yearly pattern after 1990, so that the yearly pattern of an estimate is governed primarily by Adj_HP and CFM data, rather than the CO data.

## 5.ANALYSES OF CASE-CONTROL DATA

The case-control study is nested within the cohort study reported by Attfield *et al*.^17^ and described in Silverman *et al*.^18^ Briefly, from the cohort study of 12,315 workers from eight mines, 198 deaths from lung cancer out of a total of 217^18^ deaths observed were selected as cases for the case-control study. Each case was matched to up to four controls who were alive when the case subject died, resulting in a total of 666 controls. Not all of those controls were unique, as some cohort members served as controls for more than one case, and cases were eligible to serve as controls for cases that died earlier. Controls were individually matched to each case subject by mine, sex, race/ethnicity, and birth year (within five years). Living controls and next of kin of lung cancer cases and ill or deceased controls were interviewed to collect information about the subject’s demographics, smoking history, occupational history, medical history (of both the subject’s and his/her family), and diet. Although responses from next of kin for cases and from subjects themselves for many controls can be subject to differential reporting errors, there is little evidence that this was a serious problem in the present case. E.g., Silverman *et al*. reported similar percentages in various smoking categories among controls from direct versus next-of-kin interviews. Silverman *et al*.^18^ reported analyses involving (1) all cases and controls, (2) only surface-only workers, and (3) only ever-underground workers. This latter group included workers who always worked underground as miners, and workers who worked both on the surface and underground. In our analyses we used these groupings, and also conducted separate analyses for the workers who always worked underground and did not work on the surface.

### 5.1.Statistical Analysis

Our statistical analysis of the case-control data is similar to that of Silverman *et al*.^18^,^19^ Cut points for exposure were selected to achieve approximately equal numbers of cases in each of four quartiles of exposure. Odds ratios (ORs) and 95% confidence intervals (CIs) were estimated for each quartile by conditional logistic regression. Conditional logistic regression was also used to implement two trend tests. One (T1) assigned the average estimated REC exposure in each of the four quartiles to all members of the quartile (same as Silverman *et al*., except that they used medians instead of averages^18^), and the other (T2) used the individual estimated REC exposures. The T2 trend test was unique to our analyses. As in Silverman *et al*.^18^ all reported *p*-values are from two-sided statistical tests.

### 5.2.Adjusting for Potential Confounding Covariates, Including Radon

In addition to adjusting for the interaction of smoking and primary location of employment (surface-only or ever-underground), Silverman *et al*.^18^ also adjusted for employment in certain high-risk occupations for lung cancer for at least 10 years, and history of certain nonmalignant respiratory diseases diagnosed at least five years before death (if a case) or death of the matched case (if a control). Although the exact method of selecting this particular set of covariates was not specified, Silverman *et al*.^18^ stated that they also considered a number of other factors as potential confounders, including cumulative exposure to radon, but none of those were “included in the final models because they had little or no impact on odds ratios (i.e., inclusion of these factors in the final models changed point estimates for diesel exposure by ≤10%).” However, when we reproduced the Silverman *et al*. analysis, we could not verify this statement. Table1 compares the results for all workers in Silverman *et al*. (Silverman *et al*. Table3, which contains their “primary estimates of risk”^18^) with the identical analysis, except that cumulative radon exposures were also adjusted. This table shows that, as opposed to the claim by Silverman *et al*., adjusting for radon attenuated many of the ORs, changing a number of them by more than 10%. Also, the slope estimates when adjusting for radon all were smaller by more than 10% (24% to 105%) from those when not adjusting for radon. Overall, the evidence for an effect of REC upon lung cancer mortality was much weaker after adding adjustment for radon (Table1).

**I:** Comparison of Results for Tests of Effect of DEMS_REC1 on Lung Cancer Odds Ratios (ORs) in Table 3 of Silverman *et al*.^18^ Based on All Subjects with the Identical Analysis Except Adjustment for Radon Exposure Was Added

			Table 3 of Silverman *et al*.^18^			After Adding Adjustment for Radon^a^		
Exposure Metric	Cases	Controls	OR (95% CI)	*P*_trend_	Slope^b^ 95% CI	OR (95% CI)	*P*_trend_	Slope 95% CI
Quartiles of average								
REC intensity,								
unlagged (μg/m^3^)								
0 to <1	49	166	1.0 (referent)	0.03	0.0046	1.0 (referent)	0.10	0.0035
1 to <32	50	207	1.03 (0.5 to 2.09)		(0.00055,0.0086)	1.03 (0.5 to 2.1)		(−0.00068,0.0076)
32 to < 98	49	145	1.88 (0.76 to 4.66)			1.58 (0.63 to 3.98)		
≥98	50	148	2.4 (0.89 to 6.47)			1.96 (0.71 to 5.37)		
Quartiles of average								
REC intensity, lagged 15 years								
(μg/m^3^)								
0 to < 1	47	190	1.0 (referent)	0.06	0.0047	1.0 (referent)	0.21	0.0033
1 to < 6	52	187	1.11 (0.59 to 2.07)		(−0.00024,0.0097)	1.11 (0.59 to 2.08)		(−0.0018,0.0085)
6 to < 57	49	141	1.9 (0.9 to 3.99)			1.58 (0.73 to 3.41)		
≥57	50	148	2.28 (1.07 to 4.87)			1.83 (0.83 to 4.05)		
Quartiles of cumulative								
REC, unlagged								
(μg/m^3^-y)								
0 to < 19	49	151	1.0 (referent)	0.08	0.000251	1.0 (referent)	NT^c^	−0.0000090
19 to < 246	50	214	0.87 (0.48 to 1.59)		(−0.000033,0.00054)	0.95 (0.52 to 1.76)		(−0.00037,0.00035)
246 to < 964	49	147	1.5 (0.67 to 3.36)			1.44 (0.64 to 3.24)		
≥964	50	154	1.75 (0.77 to 3.97)			1.16 (0.47 to 2.86)		
Quartiles of cumulative								
REC, lagged 15 years								
(μg/m^3^-y)								
0 to <3	49	158	1.0 (referent)	0.001	0.0010	1.0 (referent)	0.03	0.00076
3 to <72	50	228	0.74 (0.4 to 1.38)		(0.00041,0.0016)	0.75 (0.4 to 1.41)		(0.000071,0.0015)
72 to <536	49	157	1.54 (0.74 to 3.2)			1.45 (0.68 to 3.05)		
≥536	50	123	2.83 (1.28 to 6.26)			2.26 (0.94 to 5.46)		
Duration of exposure								
unexposed	48	165	1.0 (referent)	0.04	0.028	1.0 (referent)	NT	−0.0013
<5 years	51	169	1.16 (0.53 to 2.55)		(0.00085,0.055)	1.38 (0.61 to 3.09)		(−0.040,0.037)
5 to <10 years	20	95	0.88 (0.38 to 2.03)			0.9 (0.39 to 2.09)		
10 to <15 years	31	107	0.93 (0.39 to 2.21)			0.82 (0.34 to 1.98)		
≥15 years	48	130	2.09 (0.89 to 4.9)			1.32 (0.5 to 3.51)		

a Silverman *et al*.^18^ adjusted for first respiratory disease (excluding asthma and pneumonia) diagnosed before case death, history of an earlier high-risk job for lung cancer for at least 10 years, and (smoking × location [all surface work, versus some underground work]) interaction. Results labeled “after adding adjustment for radon” adjusted for each of these variables, plus radon, adjusted using cumulative radon exposure (no lag).

b Slope and 95% CI were not provided in Silverman *et al*.^18^

c NT indicates a negative trend.

Radon exposures were quantified in DEMS as mine- and year-specific estimates in working levels. However, the methods used to develop those estimates were not well-described in the original publications by the DEMS investigators. The values assigned to underground work by year and recorded in the DEMS data set were all either 0.01 or 0.02 working levels. Recognizing that radon is a well-established human lung carcinogen,^24^ we felt it was important to evaluate its influence. Consequently, we conducted most analyses in two ways, adjusting for two different sets of potential confounding covariates: one set that included radon; and one set that did not. We determined these two sets of potential confounding covariates (“with radon” and “without radon”) by forward, backward, and step-wise regressions using the SAS^©^ default options.

The set of potential confounding covariates that did not include radon (“without radon”) included:

1. First respiratory disease (excluding asthma and pneumonia): none, diagnosed less than five years before case death, five years or more before case death, or unknown.
2. Smoking status: never smoker, occasional smoker, former smoker <1 pack per day, former smoker 1 to <2 packs per day, former smoker ≥ 2 packs per day, current smoker <1 pack per day, current smoker 1 to <2 packs per day, current smoker ≥ 2 packs per day, or unknown.
3. Body mass: body mass index grouped according to WHO categories, or unknown.
4. Numbers of smokers in residence at any childhood and adulthood homes: 0, 1, ≥ 2, or unknown.

The set of potential confounding covariates that included radon (“with radon”) included the covariates listed above, and also included:

1. Cumulative radon exposure derived from estimated working levels multiplied by months at each job, summed across jobs (analyzed as a continuous variable).
2. Family history of lung cancer: no, yes, or unknown.
3. High-risk jobs of 10 or more years’ duration: no, yes, or unknown.

In addition to the covariates listed above, other covariates that we tested as possible confounding variables included those related to physical activity, exposure to respirable dust, exposure to asbestos, exposure to polycyclic aromatic hydrocarbons, exposure to silica, cigar smoking, pipe smoking, and level of education. With radon included as a possibility, all three covariate selection methods gave the same seven covariates listed above. With radon excluded as a possibility, results were not as uniform, but all three methods included the four covariates listed above in the final lists. In addition to those covariates, our analyses of all workers also adjusted for the location of work (surface-only or ever-underground). We considered adjusting for the interaction of location and smoking (as Silverman *et al*.^18^) but in a test for such interaction, adjusting for “without radon” covariates, and also adjusting for quartiles of cumulative DEMS_REC1 lagged 15 years, the interaction term was not quite significant (*p* = 0.08) and, more importantly, tests for an effect of REC exposure appeared to be little impacted by whether or not smoking was entered as a main effect and in an interaction term or only as a main effect. Therefore, we did not include a term for the interaction of location and smoking.

The smoking variable used in our analyses was defined in the same way as in Silverman *et al*.,^18^ but differed very slightly from theirs due to differences in the interpretation of smoking categories. However, these minor differences did not have any material effect on the results.

## 6.RESULTS

Using the DEMS case-control data provided to us by the DEMS investigators, we were able to reproduce all the quantitative results reported in Silverman *et al*.^3^ This gave us confidence that we were working with the same basic data set as used by Silverman *et al*. After completing this work, we set about to determine the effect of alternative REC measures and alternative ways of adjusting for potentially confounding variables, especially radon.

Our replication of the results of Silverman *et al*.^18^ included replicating their analysis of the effect of cigarette smoking on lung cancer mortality overall and cross-classified by location of employment (i.e., surface-only and ever-underground). As expected, a substantial effect of cigarette smoking (current or former) was observed. Silverman *et al*.,^18^ Table2, reported that among all subjects, irrespective of work location, OR = 5.4, 95% CI = 2.2 to 13.1, for former smokers of ≥ 2 packs per day versus never smokers, and OR = 12, 95% CI = 5.6 to 28, for current smokers of ≥ 2 packs per day versus never smokers. Silverman *et al*.^18^ also reported that among never smokers, risks of lung cancer mortality were similar between underground and surface-only workers after adjustment for 15-year lagged cumulative REC (ever-underground versus surface-only OR = 0.90, 95% CI = 0.26 to 3.1), “suggesting that the risk experienced by surface-only workers was mainly due to smoking.” We replicated all of those results.

**II:** Odds Ratios (ORs) and Trend Tests Based on DEMS_REC1 Estimates, for All Subjects

					“Without Radon“ Adjustment					"With Radon" Adjustment			
Exposures					T1 Trend		T2 Trend			T1 Trend		T2 Trend	
Range	Average	Cases	Controls	OR (95% CI)	*p*-value	Slope 95% CI	*p*-value	Slope 95% CI	OR (95% CI)	*p*-value	Slope 95% CI	*p*-value	Slope 95% CI
Intensity exposures (μg/m^3^) unlagged													
0 to < 1.4	0.87	49	166	1.0 (referent)	0.04	0.0040	0.09	0.0029	1.0 (referent)	0.11	0.0033	0.16	0.0025
1.4 to < 32.3	7.77	50	207	1.40 (0.69 to 2.85)		(0.00012,0.0078)		(−0.00048,0.0062)	1.32 (0.64 to 2.76)		(−0.00074,0.0074)		(−0.00099,0.0059)
32.3 to < 98.4	58.25	49	145	2.47 (1.00 to 6.08)					1.92 (0.75 to 4.91)				
≥98.4	181.38	50	148	3.16 (1.15 to 8.70)					2.55 (0.89 to 7.31)				
Intensity exposures (μg/m^3^) lagged 5 years													
0 to < 0.9	0.68	46	125	1.0 (referent)	0.53	0.0012	0.49	0.0012	1.0 (referent)	0.75	0.00068	0.65	0.00083
0.9 to < 21.4	4.24	53	229	0.57 (0.30 to 1.09)		(−0.0026,0.0051)		(−0.0022,0.0047)	0.57 (0.29 to 1.11)		(−0.0035,0.0049)		(−0.0027,0.0044)
21.4 to < 80.2	48.55	49	145	1.11 (0.47 to 2.63)					0.81 (0.32 to 2.00)				
≥80.2	169.95	50	167	0.93 (0.37 to 2.33)					0.75 (0.28 to 2.00)				
Intensity exposures (μg/m^3^) lagged 15 years													
0 to < 0.9	0.2	46	179	1.0 (referent)	0.06	0.0043	0.04	0.0039	1.0 (referent)	0.19	0.0032	0.08	0.0034
0.9 to < 5.8	1.85	53	198	1.38 (0.71 to 2.67)		(−0.00024,0.0087)		(0.00019,0.0077)	1.28 (0.65 to 2.53)		(−0.0015,0.0078)		(−0.00044,0.0073)
5.8 to < 57.0	31.73	49	141	2.20 (1.00 to 4.86)					1.41 (0.60 to 3.32)				
≥57.0	143.09	50	148	2.67 (1.20 to 5.94)					1.89 (0.80 to 4.44)				
Cumulative exposures (μg/m^3^-y) unlagged													
0 to < 18.7	8.48	49	151	1.0 (referent)	0.08	0.0002	0.10	0.00015	1.0 (referent)	0.78	0.000043	NT	−0.000050
18.7 to < 245.8	71.35	50	214	0.79 (0.43 to 1.47)		(−0.000025,0.00044)		(−0.000032,0.00034)	0.87 (0.46 to 1.65)		(−0.00025,0.00034)		(−0.00032,0.00022)
245.8 to < 963.9	506.21	49	147	1.78 (0.77 to 4.10)					1.71 (0.72 to 4.03)				
≥963.9	2430.71	50	154	1.91 (0.82 to 4.45)					1.38 (0.54 to 3.56)				
Cumulative exposures (μg/m^3^-y) lagged 5 years													
0 to < 11.6	5.35	49	130	1.0 (referent)	0.04	0.00029	0.06	0.00020	1.0 (referent)	0.41	0.00015	NT	−0.0000023
11.6 to < 167.5	48.57	50	222	0.64 (0.35 to 1.17)		(0.000018,0.00057)		(−0.000010,0.00041)	0.67 (0.36 to 1.24)		(−0.00020,0.00049)		(−0.00030,0.00030)
167.5 to < 880.2	454.08	49	166	1.00 (0.44 to 2.26)					0.87 (0.37 to 2.04)				
≥880.2	2177.08	50	148	1.46 (0.62 to 3.44)					1.09 (0.41 to 2.89)				
Cumulative exposures (μg/m^3^-y) lagged 15 years													
0 to < 3.4	0.42	49	158	1.0 (referent)	0.0006	0.00082	0.06	0.00035	1.0 (referent)	0.02	0.00064	0.72	0.000080
3.4 to < 71.6	20.38	50	228	0.79 (0.41 to 1.52)		(0.00035,0.0013)		(−0.000015,0.00071)	0.80 (0.41 to 1.56)		(0.000090,0.0012)		(−0.00036,0.00052)
71.6 to < 535.7	270.45	49	157	1.62 (0.75 to 3.49)					1.33 (0.59 to 3.00)				
≥535.7	1385.17	50	123	3.24 (1.40 to 7.55)					2.46 (0.94 to 6.47)				
Duration of exposure, unlagged, years													
0 to <5.3	2.75	49	123	1.0 (referent)	NT	−0.012	NT	−0.0081	1.0 (referent)	SNT	−0.035	SNT	−0.029
5.3 to <12.6	9.51	49	173	0.90 (0.51 to 1.59)		(−0.035,0.010)		(−0.030,0.014)	0.73 (0.40 to 1.33)		(−0.062,-0.0075)		(−0.055,-0.0020)
12.6 to <21.5	16.77	50	157	1.02 (0.58 to 1.80)					0.76 (0.41 to 1.40)				
≥21.5	28.74	50	213	0.70 (0.38 to 1.29)					0.38 (0.18 to 0.78)				

NT means negative trend (not significant), SNT means significant (*p* < 0.05) negative trend.

T1 trend is a trend test that assigns all members of an exposure quartile the average exposure in the quartile (similar to Silverman *et al*.^18^).

T2 trend is a trend test that uses each subject’s individual estimated REC exposure. The trend slopes are in inverse units of exposures.

Trend slopes are in reciprocal units of those of exposures.

Table2 shows results from analyses applied to all subjects utilizing the DEMS_REC1 estimates that were also used in the original case-control analysis by Silverman *et al*.^18^ These analyses differ from those reported in Silverman *et al*.’s Table3 only in terms of the variables adjusted for in the analyses. Table2 presents two sets of analyses, one that adjusts for “with radon” variables, and one that adjusts for “without radon” variables. In the analyses adjusted for “without radon” variables, trends defined in terms of average REC intensity, average intensity lagged 15 years, cumulative REC, cumulative REC lagged 15 years, and duration of exposure are all statistically significant or nearly so (T1 *p* ≤ 0.08), and the most significant is from cumulative REC lagged 15 years (T1 trend slope = 0.00082 (μg/m^3^-y)^−1^, 95% CI: 0.00035, 0.0013, T1 *p*  =  0.0006) just as was shown in Table3 of Silverman *et al*. (*p* = 0.001). Thus, despite adjusting for a different set of covariables than Silverman *et al*., our analyses that adjust for “without radon” variables show results very similar to those shown in Table3 of Silverman *et al*. (also reported herein in Table1).

**III:** Odds Ratios (ORs) and Trend Tests Based on Cumulative Exposure Lagged 15 Years, for All Subjects

					"Without Radon" Adjustment					"With Radon" Adjustment			
Exposures					T1 Trend		T2 Trend			T1 Trend		T2 Trend	

Range (μg/m^3^-y)	Average (μg/m^3^-y)	Cases	Controls	OR (95% CI)	*p*-value	Slope 95% CI	*p*-value	Slope 95% CI	OR (95% CI)	*p*-value	Slope 95% CI	*p*-value	Slope 95% CI
**DEMS_REC1**													
0 to < 3.4	0.42	49	158	1.0 (referent)	0.0006	0.00082	0.06	0.00035	1.0 (referent)	0.02	0.00064	0.72	0.000080
3.4 to < 71.6	20.38	50	228	0.79 (0.41 to 1.52)		(0.00035,0.0013)		(−0.000015,0.00071)	0.80 (0.41 to 1.56)		(0.000090,0.0012)		(-0.00036,0.00052)
71.6 to < 535.7	270.45	49	157	1.62 (0.75 to 3.49)					1.33 (0.59 to 3.00)				
≥535.7	1385.17	50	123	3.24 (1.40 to 7.55)					2.46 (0.94 to 6.47)				
**DEMS_REC2**													
0 to < 3.4	0.38	49	158	1.0 (referent)	0.0008	0.00090	0.06	0.00040	1.0 (referent)	0.05	0.00063	0.65	0.00012
3.4 to < 80.1	23.03	50	227	0.84 (0.44 to 1.59)		(0.00037,0.0014)		(−0.000017,0.00083)	0.82 (0.42 to 1.60)		(−0.0000060,0.0013)		(−0.00039,0.00063)
80.1 to < 457.6	235.19	49	154	1.62 (0.75 to 3.51)					1.38 (0.61 to 3.12)				
≥457.6	1,194.05	50	127	3.14 (1.36 to 7.27)					2.29 (0.87 to 6.07)				
**DEMS_REC3**													
0 to < 3.4	0.40	49	159	1.0 (referent)	0.001	0.00076	0.02	0.00047	1.0 (referent)	0.07	0.00055	0. 76	0.000092
3.4 to < 88.8	24.18	50	225	0.85 (0.44 to 1.61)		(0.00030,0.0012)		(0.000085,0.00086)	0.82 (0.42 to 1.61)		(−0.000045,0.0011)		(−0.00049,0.00068)
88.8 to < 656.0	348.68	49	155	1.77 (0.83 to 3.78)					1.51 (0.68 to 3.36)				
≥656.0	1,415.25	50	127	3.17 (1.35 to 7.42)					2.35 (0.85 to 6.52)				
**REC1**													
0 to < 6.4	0.78	49	157	1.0 (referent)	0.01	0.00047	0.02	0.00035	1.0 (referent)	0.65	0.00012	NT	−0.000030
6.4 to < 96.7	35.34	50	214	0.80 (0.43 to 1.49)		(0.00012,0.00083)		(0.000062,0.00065)	0.75 (0.39 to 1.44)		(−0.00040,0.00063)		(−0.00053,0.00047)
96.7 to < 772.7	380.2	49	162	1.58 (0.73 to 3.41)					1.29 (0.57 to 2.92)				
≥772.7	1782.58	50	133	2.49 (1.06 to 5.85)					1.35 (0.44 to 4.12)				
**REC2**													
0 to < 6.3	0.68	49	159	1.0 (referent)	0.002	0.00058	0.05	0.00030	1.0 (referent)	0.17	0.00035	NT	−0.000090
6.3 to < 99.2	32.81	50	217	0.75 (0.39 to 1.44)		(0.00022,0.00094)		(−0.0000038,0.00061)	0.70 (0.36 to 1.37)		(−0.00015,0.00084)		(−0.00053,0.00035)
99.2 to < 752.9	385.64	49	167	1.73 (0.78 to 3.83)					1.47 (0.63 to 3.44)				
≥752.9	1798.46	50	123	3.03 (1.25 to 7.33)					2.04 (0.67 to 6.24)				
**REC3**													
0 to < 0. 6	0.08	49	194	1.0 (referent)	0.19	0.00033	0.60	0.000090	1.0 (referent)	0.68	0.00011	NT	−0.00013
0.6 to < 17.8	7.54	50	169	1.36 (0.71 to 2.61)		(−0.00016,0.00082)		(−0.00024,0.00042)	1.25 (0.63 to 2.48)		(−0.00041,0.00064)		(−0.00050,0.00024)
17.8 to < 224. 4	71.96	49	150	1.87 (0.90 to 3.88)					1.45 (0.66 to 3.16)				
≥224.4	1182.65	50	153	2.31 (1.01 to 5.27)					1.55 (0.63 to 3.84)				
**REC4**													
0 to < 4.9	0.71	49	168	1.0 (referent)	0.04	0.00041	0.09	0.00027	1.0 (referent)	0.74	0.000084	NT	−0.000080
4.9 to < 70.4	26.65	50	216	0.84 (0.44 to 1.59)		(0.000011,0.00081)		(−0.000044,0.00058)	0.80 (0.41 to 1.55)		(−0.00041,0.00058)		(−0.00049,0.00033)
70.4 to < 498.4	243.34	49	143	2.12 (0.98 to 4.58)					1.67 (0.73 to 3.81)				
≥498.4	1522.1	50	139	2.45 (1.05 to 5.76)					1.50 (0.54 to 4.17)				
**REC5**													
0 to <7.4	0.77	49	158	1.0 (referent)	0.01	0.00044	0.05	0.00026	1.0 (referent)	0.46	0.00017	NT	−0.000080
7.4 to <126.2	40.37	50	218	0.78 (0.41 to 1.47)		(0.00012,0.00077)		(−0.0000023,0.00053)	0.72 (0.37 to 1.39)		(−0.00028,0.00061)		(−0.00046,0.00030)
126.2 to <848.2	449.03	49	156	1.78 (0.80 to 3.93)					1.39 (0.60 to 3.24)				
≥848.2	1994.92	50	134	2.66 (1.11 to 6.39)					1.52 (0.50 to 4.56)				
**REC6**													
0 to <2.8	0.61	49	181	1.0 (referent)	0.05	0.00054	0.08	0.00035	1.0 (referent)	0.72	0.00012	NT	−0.0000035
2.8 to <50.6	20.47	50	197	1.09 (0.60 to 2.0)		(0.0000049,0.0011)		(−0.000048,0.00074)	1.07 (0.57 to 2.0)		(−0.00053,0.00077)		(−0.00051,0.00050)
50.6 to <388.0	158.27	49	157	1.84 (0.90 to 3.8)					1.35 (0.62 to 2.94)				
≥388.0	1156.89	50	131	2.56 (1.1 to 5.9)					1.43 (0.52 to 3.94)				

NT means negative trend (not significant), SNT means significant (*p* < 0.05) negative trend.

T1 trend is a trend test that assigns all members of an exposure quartile the average exposure in the quartile (similar to Silverman *et al*.^18^).

T2 trend is a trend test that uses each subject’s individual estimated REC exposure. The trend slopes are in units of (μg/m^3^-y) ^−1^.

Table2 also contains results for average REC intensity lagged five years and cumulative REC lagged five years, which were not reported by Silverman *et al*.^18^,^19^ We conducted these analyses because that lag was preferred in other epidemiological analyses of DEE and lung cancer.^25^,^26^

The results for duration of exposure in Table2 differ greatly from the corresponding results in Silverman *et al*.^18^ Whereas Silverman *et al*. found a significant (*p* = 0.043) positive trend with increasing duration of exposure, Table2 shows all negative trends with duration of exposure, which are significant (*p*<0.05) when adjusting for radon. In our analysis, we divided the subjects by quartiles of duration of exposure, just as was done for other exposure measures, whereas Silverman *et al*. divided the workers into five groups by years of exposure, and included a group of 213 above-ground workers (out of a total of 328 above-ground workers) who were assumed to be unexposed, although virtually all workers had some assigned exposure. Silverman *et al*. state that those workers assigned zero exposure “had either negligible or bystander exposure to REC,” but we could not determine how these designations were determined.

The results from adjusting for “with radon” variables in Table2 show much less evidence of an association between REC exposure and lung cancer mortality. In fact, the only analysis reported in this table that adjusted for radon and showed a significant trend was the T1 trend for cumulative exposure lagged 15 years (T1 *p* = 0.02, T1 slope = 0.00064 (μg/m^3^-y)^−1^, 95% CI: 0.000090, 0.0012, T2 *p* = 0.72, T2 slope: 0.00008 (μg/m^3^-y)^−1^, 95% CI: −0.00036, 0.00052), which is a substantially weaker and less significant result than was obtained when not adjusting for radon (T1 *p* = 0.0006, T1 slope = 0.00082 (μg/m^3^-y)^−1^, 95% CI: 0.00035, 0.0013, T2 *p* = 0.06, T2 slope = 0.00035 (μg/m^3^-y)^−1^, 95% CI: −0.000015, 0.00071). Apart from cumulative exposure lagged 15 years, none of the other exposure metrics showed a significant trend when adjusting for “with radon” variables. Thus, adjusting for “with radon” variables resulted in considerably diminished evidence for an association between REC and lung cancer mortality.

The most significant results in Table2, in both analyses that adjusted for radon and those that did not, were for cumulative exposure lagged 15 years. In each of the alternative measures of REC we have studied, this has generally been the case. Consequently, in our presentation of the results based on different measures of REC, we focused on cumulative exposure lagged 15 years.

Table3 shows results from applying the different REC measures to data on all workers, irrespective of work location, to calculate trends associated with cumulative exposure to REC lagged 15 years. It is apparent that adjusting for radon makes a very substantial difference in the evidence for an association between REC and lung cancer mortality. Whereas the trends from all of the REC measures, except REC3, are significant or nearly so when adjusting for “without radon” variables, only the three DEMS REC estimates (DEMS_REC1, DEMS_REC2, and DEMS_REC3) are significant or nearly so when adjusting for “with radon” variables, and then only based on T1 trend. Based on T2 trend, every one of the other REC estimates shows a negative trend.

Table4 contains the same analyses as in Table3 except that the miners are restricted to those who ever worked underground. This subgroup includes individuals who always worked underground as well as those who worked both underground and on the surface. Adjusting for “without radon” variables, the T1 trends for several of the REC exposures are significant or nearly so, but, again, the T2 trends are not. Adjusting for “with radon” variables, only the DEMS_REC1 T1 trend is significant (T1 *p* = 0.05, T1 slope = 0.00067, 95% CI: −0.000012, 0.0014), but the corresponding T2 trend is negative, along with a number of both T1 and T2 trends for other REC measures.

**IV:** Odds Ratios (ORs) and Trend Tests Based on Cumulative Exposure Lagged 15 Years, for Subjects Who Ever Worked Underground

					“Without Radon” Adjustment					"With Radon" Adjustment			
Exposures					T1 Trend		T2 Trend			T1 Trend		T2 Trend	

Range (μg/m^3^-y)	Average (μg/m^3^-y)	Cases	Controls	OR (95% CI)	*p*-value	Slope 95% CI	*p*-value	Slope 95% CI	OR (95% CI)	*p*-value	Slope 95% CI	*p*-value	Slope 95% CI
**DEMS_REC1**													
0 to < 97.0	20.0	31	153	1.0 (referent)	0.01	0.00073	0.17	0.00028	1.0 (referent)	0.05	0.00067	NT	−0.0000040
97.0 to < 383.5	225.7	31	94	1.90 (0.78 to 4.63)		(0.00022,0.0012)		(−0.00012,0.00067)	1.82 (0.71 to 4.62)		(−0.000012,0.0014)		(−0.00058,0.00057)
383.5 to < 902.7	584.4	31	89	2.73 (1.08 to 6.88)					2.84 (0.97 to 8.28)				
≥902.7	1812.5	31	76	5.04 (1.77 to 14.30)					5.38 (1.29 to 22.57)				
**DEMS_REC2**													
0 to < 108.9	27.0	31	158	1.0 (referent)	0.05	0.00060	0.12	0.00040	1.0 (referent)	0.51	0.00029	0.79	0.000095
108.9 to < 318.2	209.6	31	90	2.73 (1.14 to 6.55)		(−0.000011,0.0012)		(−0.00011,0.00091)	2.53 (1.02 to 6.31)		(−0.00057,0.0011)		(−0.00060,0.00079)
318.2 to < 782.0	511.3	31	80	4.21 (1.52 to 11.71)					3.94 (1.20 to 12.97)				
≥782.0	1483.0	31	84	4.19 (1.43 to 12.26)					3.40 (0.78 to 14.87)				
**DEMS_REC3**													
0 to < 157.1	34.1	31	160	1.0 (referent)	0.02	0.00064	0.14	0.00035	1.0 (referent)	0.21	0.00052	0.84	−0.000080
157.1 to < 521.7	332.4	31	94	2.72 (1.16 to 6.37)		(0.00011,0.0012)		(−0.00011,0.00081)	2.66 (1.09 to 6.48)		(−0.00030,0.0013)		(−0.00087,0.00071)
521.7 to < 957.4	692.5	31	78	3.21 (1.15 to 8.98)					2.95 (0.89 to 9.76)				
≥957.4	1792.3	31	80	4.67 (1.61 to 13.57)					4.24 (0.87 to 20.71)				
**REC1**													
0 to < 158.6	36.4	31	145	1.0 (referent)	0.20	0.00030	0.18	0.00027	1.0 (referent)	NT	−0.00024	NT	−0.00020
158.6 to < 649.2	372.4	31	113	2.03 (0.87 to 4.69)		(−0.00016,0.00075)		(−0.00013,0.00067)	1.77 (0.73 to 4.28)		(−0.00097,0.00049)		(−0.00094,0.00054)
649.2 to < 1287.8	883.3	31	73	3.96 (1.45 to 10.80)					2.57 (0.75 to 8.85)				
≥1287.8	2285.9	31	81	2.58 (0.81 to 8.25)					1.02 (0.17 to 6.36)				
**REC2**													
0 to < 165.3	33.4	31	146	1.0 (referent)	0.12	0.00033	0.26	0.00023	1.0 (referent)	NT	−0.000010	NT	−0.00025
165.3 to < 556.5	347.6	31	105	2.14 (0.91 to 5.02)		(−0.000087,0.00074)		(−0.00017,0.00064)	1.95 (0.79 to 4.80)		(−0.00065,0.00063)		(−0.00065,0.00063)
556.5 to < 1101.4	773.8	31	75	4.13 (1.43 to 11.93)					3.23 (0.96 to 10.86)				
≥1101.4	2157.7	31	86	3.07 (1.07 to 8.84)					1.84 (0.38 to 8.88)				
**REC3**													
0 to < 16.6	3.1	31	148	1.0 (referent)	0.11	0.00042	0.76	0.000061	1.0 (referent)	0.67	0.00013	NT	−0.00025
16.6 to < 95.6	34.6	31	77	2.62 (0.92 to 7.51)		(−0.000091,0.00093)		(−0.00033,0.00045)	2.26 (0.75 to 6.84)		(−0.00047,0.00073)		(−0.00076,0.00026)
95.6 to < 693.9	319.2	31	99	2.64 (0.97 to 7.18)					2.00 (0.67 to 6.00)				
≥693.9	1747.6	31	88	4.26 (1.30 to 13.95)					2.55 (0.60 to 10.82)				
**REC4**													
0 to < 101.6	20.6	31	145	1.0 (referent)	0.09	0.00041	0.38	0.00019	1.0 (referent)	0.69	0.00013	NT	−0.00026
101.6 to < 329.8	216.1	31	100	2.15 (0.86 to 5.40)		(−0.000061,0.00089)		(−0.00023,0.00062)	1.94 (0.75 to 5.04)		(−0.00051,0.00077)		(−0.00089,0.00037)
329.8 to < 964.2	599.6	31	83	3.64 (1.30 to 10.18)					2.85 (0.91 to 8.89)				
≥964.2	2036.4	31	84	3.92 (1.22 to 12.56)					2.46 (0.53 to 11.38)				
**REC5**													
0 to < 231.4	51.3	31	154	1.0 (referent)	0.08	0.00033	0.22	0.00022	1.0 (referent)	0.82	0.000065	NT	−0.00017
231.4 to < 711.9	431.8	31	96	2.73 (1.14 to 6.58)		(−0.000036,0.00070)		(−0.00013,0.00058)	2.60 (1.04 to 6.52)		(−0.00049,0.00062)		(−0.00076,0.00042)
711.9 to <1241.4	936.1	31	75	2.97 (1.09 to 8.12)					2.30 (0.68 to 7.74)				
≥1241.4	2500.4	31	87	3.55 (1.24 to 10.14)					2.16 (0.45 to 10.31)				
**REC6**													
0 to <64.7	14.7	31	152	1.0 (referent)	0.06	0.00060	0.32	0.00026		0.44	0.00033	NT	−0.00019
64.7 to <204.9	117.6	31	85	2.51 (1.00 to 6.33)		(−0.000012,0.00122)		(−0.00025,0.00076)	2.40 (0.92 to 6.27)		(−0.00050,0.0012)		(−0.00092,0.00054)
204.9 to <717.0	429.4	31	98	3.22 (1.21 to 8.54)					2.60 (0.87 to 7.78)				
≥717.0	1570.0	31	77	4.50 (1.42 to 14.24)					3.37 (0.73 to 15.68)				

NT means negative trend (not significant), SNT means significant (*p* < 0.05) negative trend.

T1 trend is a trend test that assigns all members of an exposure quartile the average exposure in the quartile (similar to Silverman *et al*.^(18)^).

T2 trend is a trend test that uses each subject’s individual estimated REC exposure. The trend slopes are in units of (μg/m^3^- y)−^1^.

Table5 contains results of the same analyses as reported in Tables3 and 4, except that the analyses were restricted to workers who worked only in underground jobs. None of the REC measures showed statistically significant evidence of a lung cancer effect related to REC, and five out of the six T2 trends based on our alternative REC exposures were negative, whether or not radon was controlled. Thus, there is progressively less evidence of a lung cancer effect related to REC as the analysis is restricted more completely to underground workers—the workers with the highest exposures to DEE. The restricted analyses eliminated all controls for an omitted case, as well as every case for which all controls had been eliminated. Thus, the numbers of cases and controls were reduced as the analyses progressed from all workers (Table3), based on 198 lung cancer deaths and 666 controls; to the analyses restricted to only-underground workers (Table4), based on 124 cases and 412 controls; to the analyses restricted to workers who only worked underground (Table5), based on 58 cases and 97 controls. The decrease in statistical significance (i.e., larger *p*-values) as the analysis is restricted to fewer workers could be due, in part, to a decrease in power to detect an effect. However, this does not appear to be the complete explanation. Among the six measure of REC that were most significantly related to REC analyses of all workers (T1 *p* ≤ 0.01), the corresponding T1 trend slopes, when restricted to those who always worked underground (Table5), were reduced by fractions ranging from 1.5 to 3.7. Thus, there is some evidence suggesting a weaker effect of REC if analyses are restricted to workers who only worked underground.

**V:** Odds Ratios (ORs) and Trend Tests Based on Cumulative Exposure Lagged 15 Years, for Subjects Who Only Worked Underground

					“Without Radon” Adjustment					“With Radon” Adjustment			
Exposures					T1 Trend		T2 Trend			T1 Trend		T2 Trend	

Range (μg/m^3^-y)	Average (μg/m^3^-y)	Cases	Controls	OR (95% CI)	*p*-value	Slope 95% CI	*p*-value	Slope 95% CI	OR (95% CI)	*p*-value	Slope 95% CI	*p*-value	Slope 95% CI
**DEMS_REC1**													
0 to < 67.0	12.4	14	24	1.0 (referent)	0.26	0.00042	0.71	0.00022	1.0 (referent)	0.29	0.00067	0.95	0.000021
67.0 to < 325.3	166.7	15	22	1.00 (0.21 to 4.67)		(−0.00031,0.0011)		(−0.00065,0.0007)	1.27 (0.27 to 5.88)		(−0.00057,0.0019)		(−0.00091,0.0013)
325.3 to < 835.1	558.5	14	31	1.60 (0.25 to 10.14)					1.55 (0.18 to 13.13)				
≥835.1	2,042.5	15	20	2.51 (0.41 to 15.59)					3.41 (0.31 to 37.78)				
**DEMS_REC2**													
0 to < 105.5	31.8	14	30		0.43	0.00044	0.60	0.00027		0.24	0.00101	0.34	0.00085
105.5 to < 278.7	204.9	15	26	2.62 (0.51 to13.5)		(−0.00065,0.0015)		(−0.00075,0.0013)	2.86 (0.47 to 17.41)		(−0.00069,0.0027)		(−0.00090,0.0026)
278.7 to < 603.1	394.6	14	17	7.99 (1.15 to 55.71)					11.74 (1.17 to 118.17)				
≥603.1	1341.6	15	24	4.43 (0.69 to 28.35)					11.68 (0.65 to 210.88)				
**DEMS_REC3**													
0 to < 135.4	28.4	14	27		0.46	0.00041	0.84	0.00010		0.40	0.00073	0.57	0.00055
135.4 to < 429.7	252.4	15	22	1.86 (0.39 to 8.84)		(−0.00068,0.0015)		(−0.00087,0.0011)	1.97 (0.38 to 10.29)		(−0.00096,0.0024)		(−0.0014,0.0025)
429.7 to < 750.0	615.4	14	23	2.51 (0.39 to 16.23)					2.68 (0.30 to 23.55)				
≥750.0	1492.9	15	25	2.62 (0.42 to 16.49)					4.23 (0.28 to 63.85)				
**REC1**													
0 to < 151.1	28.9	14	25	1.0 (referent)	0.79	0.00013	NT	−0.00016	1.0 (referent)	0.58	0.00041	NT	−0.00010
151.1 to < 438.7	258.4	15	24	1.51 (0.35 to 6.48)		(−0.00082,0.0011)		(−0.0012,0.0010)	1.46 (0.29 to 7.40)		(−0.0011,0.0019)		(−0.0023,0.0020)
438.7 to < 844.4	665.0	14	21	1.02 (0.18 to 5.94)					1.10 (0.15 to 8.08)				
≥844.4	1658.1	15	27	1.40 (0.25 to 7.96)					2.20 (0.17 to 28.80)				
**REC2**													
0 to < 126.8	15.4	14	22	1.0 (referent)	0.62	0.00021	NT	−0.000080	1.0 (referent)	0.47	0.00052	NT	−0.000050
126.8 to < 380.8	247.7	15	24	1.27 (0.29 to 5.60)		(−0.00063,0.0011)		(−0.0010,0.00089)	1.23 (0.25 to 6.12)		(−0.00090,0.0019)		(−0.0018,0.0017)
380.8 to < 881.1	595.5	14	28	0.49 (0.08 to 3.06)					0.62 (0.08 to 4.52)				
≥881.1	1916.4	15	23	1.26 (0.19 to 8.53)					2.37 (0.12 to 46.69)				
**REC3**													
0 to < 12.1	1.6	14	22	1.0 (referent)	0.28	0.00053	NT	−0.00027	1.0 (referent)	0.25	0.00044	NT	−0.00023
12.1 to < 99.6	28.7	15	25	0.76 (0.09 to 6.39)		(−0.00031,0.0012)		(−0.00099,0.00053)	1.11 (0.15 to 8.18)		(−0.00043,0.0015)		(−0.0013,0.00076)
99.6 to < 700.2	302.8	14	28	0.99 (0.12 to 7.96)					0.87 (0.13 to 5.76)				
≥700.2	2221.2	15	22	2.78 (0.17 to 45.36)					2.35 (0.28 to 19.39)				
**REC4**													
0 to < 104.3	19.4	14	25	1.0 (referent)	0.20	0.0010	NT	−0.00012	1.0 (referent)	0.36	0.00044	NT	−0.00012
104.3 to < 262.5	205.8	15	24	2.77 (0.42 to 18.31)		(−0.00051,0.0014)		(−0.0012,0.0010)	2.19 (0.42 to 11.46)		(−0.00055,0.0026)		(−0.0018,0.0016)
262.5 to < 599.6	392.9	14	25	2.60 (0.24 to 27.75)					2.12 (0.29 to 15.29)				
≥599.6	1751.5	15	23	11.83 (0.38 to 364.00)					3.52 (0.43 to 29.14)				
**REC5**													
0 to < 177.8	31.0	14	24	1.0 (referent)	0.42	0.00029	0.95	0.000029	1.0 (referent)	0.25	0.00071	0.77	0.00022
177.8 to < 494.3	299.6	15	24	1.77 (0.45 to 7.06)		(−0.00042,0.0010)		(−0.00080,0.00086)	1.71 (0.37 to 7.98)		(−0.00050,0.0019)		(−0.0012,0.0016)
494.3 to < 1183.0	815.6	14	29	0.85 (0.16 to 4.64)					1.23 (0.17 to 8.71)				
≥1183.0	2346.6	15	20	2.40 (0.35 to 16.35)					6.27 (0.28 to 138.9)				
**REC6**													
0 to < 50.7	12.8	14	26	1.0 (referent)	0.66	0.00028	NT	−0.00026	1.0 (referent)	0.58	0.00057	NT	−0.00039
50.7 to < 150.1	94.9	15	24	1.12 (0.22 to 5.82)		(−0.00098,0.0015)		(−0.0014,0.00085)	1.14 (0.19 to 6.80)		(−0.0014,0.0026)		(−0.0020,0.0013)
150.1 to < 509.6	306.0	14	22	1.45 (0.26 to 8.16)					1.76 (0.27 to 11.64)				
≥509.6	1287.0	15	25	1.62 (0.24 to 11.15)					2.54 (0.14 to 45.56)				

NT means negative trend (not significant), SNT means significant (*p* < 0.05) negative trend.

T1 trend is a trend test that assigns all members of an exposure quartile the average exposure in the quartile (similar to Silverman *et al*.^18^).

T2 trend is a trend test that uses each subject’s individual estimated REC exposure. The trend slopes are in units of (μg/m^3^-y)^-1^

Summarizing, in analyses based on cumulative exposure to REC lagged 15 years, which included all workers (Table3) and adjusted for “without radon” variables, all of the nine REC measures investigated, other than REC3, showed some statistical evidence of an association with lung cancer mortality, with slopes that ranged from 0.00041 μg/m^3^-y to 0.00090 μg/m^3^-y. However, when adjusting for “with radon” variables, only the three DEMS REC measures (DEMS_REC1, DEMS_REC2, and DEMS_REC3) showed evidence of an effect of REC, and then with only the T1 trend. None of the other analyses showed an effect of REC. For each of the analyses based on all workers that adjusted for “with radon” variables (Table3), the slopes were reduced compared with those from the comparable analyses that adjusted for “without radon” variables. For analyses restricted to workers who worked only underground (Table5), none of the REC measures showed evidence of being associated with increased lung cancer mortality.

Table6 shows the effect of eliminating workers at a single mine upon the evidence for an association of REC with lung cancer mortality. These analyses used DEMS_REC1, which was the REC estimate that most consistently showed a REC effect, and were based on cumulative REC lagged 15 years, using all workers, adjusted for “with radon” variables, and using the T1 trends, which is the trend that showed an effect of REC in analyses involving all mines. This analysis indicates that the evidence for an effect of REC in these analyses is not restricted to a single mine. As discussed further below, this result differs from that from the reanalysis of the DEMS cohort data,^21^ in which both a proportional hazard regression analysis, and an analysis based on the concepts of multistage carcinogenesis, found a significant association with DEMS_REC1 in the limestone mine (mine A), but not in other mine types. Table6 indicates that a significant effect of DEMS_REC1 was still obtained in the case-control data, after eliminating data from mine A. The cohort analysis was based on the same measure of REC (DEMS_REC1) as the results in Table6, although it did not control for radon.

**VI:** Odds Ratios (ORs) Categorized by Cumulative Exposure Lagged 15 Years to DEMS_REC1 Adjusted Using “With Radon” Variables, for all Subjects After Omitting Data from a Single Mine

	Exposures					T1 Trend	
All but Mine	Range (μg/m^3^-y)	Average (μg/m^3^-y)	Cases	Controls	OR (95% CI)	*p*-value	Slope 95% CI
A	0 to < 2.0	0.10	40	120	1.0 (referent)	0.02	0.00075
	2.0 to < 25.4	10.73	41	162	0.76 (0.34, 1.72)		(0.00013,0.0014)
	25.4 to < 547.5	258.79	40	152	1.19 (0.48, 2.91)		
	≥547.5	1400.81	41	106	2.67 (0.88, 8.12)		
B	0 to < 3.4	0.42	46	147	1.0 (referent)	0.02	0.00064
	3.4 to < 76.2	20.78	47	220	0.79 (0.39, 1.57)		(0.000084,0.0012)
	76.2 to < 547.6	275.69	47	148	1.32 (0.57, 3.05)		
	≥547.6	1412.36	47	113	2.49 (0.92, 6.71)		
D	0 to < 1.6	0.02	35	120	1.0 (referent)	0.03	0.00070
	1.6 to < 49.7	18.28	36	172	0.71 (0.28, 1.8)		(0.000051,0.0014)
	49.7 to < 460.6	206.94	36	103	1.26 (0.45, 3.5)		
	≥460.6	1356.69	36	92	2.44 (0.77, 7.73)		
E	0 to < 3.4	0.44	47	152	1.0 (referent)	0.03	0.00073
	3.4 to < 56.8	17.80	47	216	0.82 (0.41, 1.63)		(0.000087,0.0014)
	56.8 to < 508.6	245.00	47	157	1.34 (0.57, 3.17)		
	≥508.6	1215.26	48	111	2.44 (0.91, 6.58)		
G	0 to < 3.4	0.44	46	145	1.0 (referent)	0.02	0.00071
	3.4 to < 82.9	22.28	47	218	0.78 (0.39, 1.54)		(0.00013,0.0013)
	82.9 to <579.2	292.28	46	151	1.38 (0.6, 3.15)		
	≥579.2	1399.97	47	110	2.69 (1, 7.24)		
H	0 to < 6.8	1.57	45	161	1.0 (referent)	0.03	0.00062
	6.8 to < 92.5	28.86	45	195	1.14 (0.58, 2.23)		(0.000045,0.0012)
	92.5 to < 563.4	295.81	45	140	2.01 (0.85, 4.73)		
	≥563.4	1428.92	45	113	3.37 (1.22, 9.3)		
I	0 to <3.4	0.33	44	133	1.0 (referent)	0.06	0.00057
	3.4 to <87.5	24.88	44	203	0.79 (0.39, 1.61)		(−0.000016,0.0012)
	87.5 to <535.7	285.13	44	139	1.18 (0.5, 2.79)		
	≥535.7	1416.91	45	116	2.11 (0.77, 5.82)		
All	0 to < 3.4	0.42	49	158	1.0 (referent)	0.02	0.00064
Mines	3.4 to < 71.6	20.38	50	228	0.79 (0.41 to 1.52)		(0.000090,0.0012)
	71.6 to < 535.7	270.45	49	157	1.62 (0.75 to 3.49)		
	≥535.7	1385.17	50	123	3.24 (1.40 to 7.55)		

## 7.DISCUSSION

**Figure 2:**
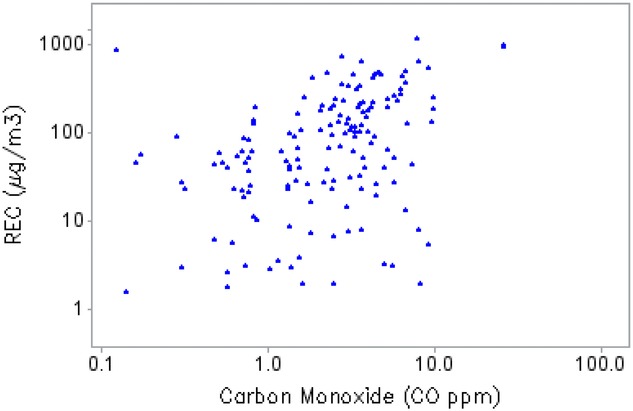
Paired CO–REC samples from the DEMS 1998–2001 survey.

All of the alternative exposure measures applied herein, except for REC6, employed the assumption that there is a reliable quantitative relationship between CO and REC emitted from diesel engines. REC6 uses only Adj_HP and CFM and does not involve CO. Fig.2 shows the paired CO–REC samples results from the DEMS 1998–2001 survey. There appears to be little visual evidence of a relationship. In analyzing those data, Stewart *et al*.^11^ found a best relationship of the form REC ∼ CO^0.58^, whereas Crump and Van Landingham^16^ found a best relationship of the form REC ∼ CO^0.3^, and Silverman *et al*.^18^ used REC exposures that assumed REC ∼ CO^1^. However, from Fig.2 it is clear that there will be a great deal of uncertainty about any presumed relationship between CO and REC.

Diesel oxidation catalysts (DOCs), an after-treatment technology for diesel-powered equipment that converts CO in the exhaust stream to CO_2_, began to be installed on diesel equipment used in mines in the 1970s and 1980s. The use of DOCs can greatly decrease ratios of CO/REC.^7^ However, no information on the extent of DOCs usage in the eight DEMS mines was used in developing any of the REC exposures. Clark *et al*.^27^ studied the relationship between particulate matter (PM) and CO in diesel engines operated under different loading conditions, and concluded that that there was no universal relationship between CO and PM, but that that the CO/PM relationship appears to be unique for each engine type and perhaps for each engine. Consequently, the assumption of a fixed quantitative relationship between CO and REC is questionable.

**Figure 3:**
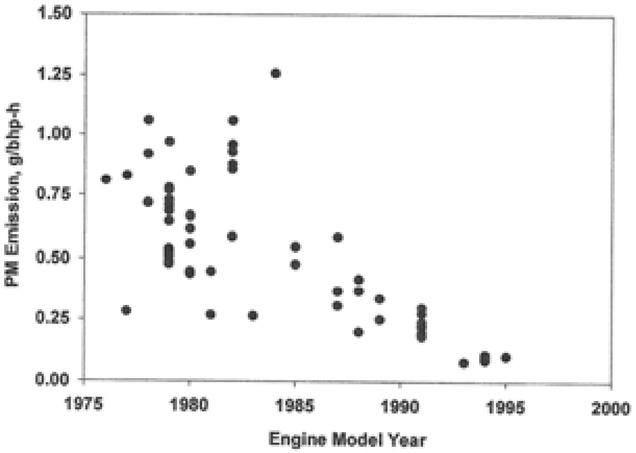
Fig. 2-20 from USEPA (2002) showing the decrease in diesel engine PM emissions per brake HP overtime.

The EPA Health Assessment for DEE^28^ includes a summary of data on the exhaust emissions of a number of pollutants, including PM and CO (Table 2.8 in that report). Fig. 2-20 from that report is reproduced here as Fig.3. It may be noted the PM emissions, expressed as grams/brake-horse-power-hour, trend downward from 0.75 in the late 1970s to 0.25 in the early 1990s. These data normalized to brake-horse-power support the validity of REC estimates, such as REC6, developed from Adj_HP and ventilation. In retrospect, it would have been appropriate to develop a REC estimate that considered the change in PM emissions over time. Recognizing that diesel engines are very durable and, thus, have a long working life in the mines, it would have been necessary to make adjustments for duration of use of specific pieces of equipment in the Adj_HP for any given mine in a particular year. The general effect of an adjustment for changes in PM (as a surrogate for REC) emission rates would be to increase the estimates of REC for earlier years relative to the 1998–2001 period, when the REC measurements were made (see Table S2 in the Supporting Information).

All of the estimates of REC, whether made using the uncertain relationships between CO and Adj_HP/CFM or between REC and Adj_HP/CFM, are based on personal measurements of REC made in 1998–2001. It is important to recall that subjects were followed through December 31, 1997, and that analyses employing a 15-year lag provided the most significant associations between DEE and lung cancer mortality. Consequently, the most relevant REC estimates involved in these analyses are for 1947–1982, which are between 15 and 50+ years earlier than any of the REC measurements that went into their determination.

We found that adjusting for radon substantially reduced the evidence for an association of REC with lung cancer mortality (Tables5). As noted earlier, this is at odds with the statement in Silverman *et al*.^18^ that several potential confounding covariates, including radon, had “little or no impact on odds ratios.” We have assumed that the radon data used by Silverman *et al*.^18^ are the same as the radon data we had access to at the NCHS Research Data Center. Radon is a known human lung carcinogen^24^ that needs to be considered as a possible confounder of associations with other potential causes of lung cancer. Uncertainty about the soundness of the radon data available from the DEMS study resulted in our conducting analyses both adjusting and not adjusting for radon.

As expected, estimates of cumulative radon exposure are correlated with duration of REC exposure in the DEMS data (Spearman correlation = 0.80, Kendall’s tau = 0.71). Nevertheless, tests for association with duration of REC exposure were more significant when adjusting for radon (Table2), so it does not appear that adjusting for radon is equivalent to adjusting for duration of REC exposure. In a study of DEE exposure and lung cancer mortality that is considered to indicate a positive association (Garshick *et al*.^26^), a relationship was found between DEE exposure and lung cancer mortality only after adjusting for duration of employment, which appears to be a credible estimate of duration of exposure in the Garshick *et al*. cohort, assuming that low background exposures while unemployed are not counted, just as they were not counted in the DEMS cohort. The Garshick *et al*.^26^ study did not involve personnel working underground, so radon was not of concern. Garshick *et al*. considered adjusting for duration of employment necessary to control for negative confounding by employment duration. Similarly, in the present study, negative associations were obtained between duration of REC exposure and lung cancer mortality. Therefore, adjusting for radon would seem to be justified in analyzing the DEMS data, either as a necessary adjustment for possible confounding by a known human carcinogen, or as a surrogate for possible negative confounding by duration of REC exposure, or, equivalently, duration of employment, as was considered to be true in Garshick *et al*.^26^

When not adjusting for radon exposure, all but one of the nine REC estimates studied was significantly associated with lung cancer mortality (Table3). When radon exposure was adjusted for, however, only the three DEMS REC estimates, but none of the other six REC estimates, provided evidence of an association of DEE with lung cancer.

Tables5 contain *p*-values computed using two trend tests. The T1 trend applies the average exposure in a quartile to every subject in the quartile (similar to the approach used in Silverman *et al*.,^18^,^19^ which used medians instead of averages), whereas the T2 trend uses each subject’s individually estimated exposure. The two tests give roughly similar results in many cases, in particular in analyses of all miners not adjusting for radon (Table3). The T2 trend generally is somewhat less significant than the T1 trend, although in many cases they lead to similar conclusions. The T1 trend involves an ad hoc decision about how to form exposure groups, whereas the T2 trend does not. Our preference would be to avoid ad hoc decisions, particularly when using the data quantitatively (as in a QRA), which argues for use of T2 trends in any QRA. However, T1 trends can be useful in determining if there is an association between DEE and lung cancer, which was the goal of Silverman *et al*.,^18^ because there is no reason why such trends would tend to show a DEE association when there is none. T2 trends can be highly influenced by a few outlying data points. For example, one of the controls had a DEMS_REC1 estimate about twice that of any case. Eliminating this control and the four other controls with the highest DEMS_REC1 estimates from the analysis (thereby eliminating only five out of 666 controls) caused the T2 *p*-value in one analysis to change from 0.12 to 0.02, whereas the corresponding T1 *p*-value changed only from 0.063 to 0.061.

Thus, although T1 trends can be useful in determining if an association exists between DEE exposure and lung cancer, those trends do not make full use of the exposure estimates available on each individual subject. Because accurate exposure determinations are equally as important as accurate determination of health outcomes in QRA, we consider that T2 trend slopes, or some similar modeling approach that does not involve aggregating exposures, perhaps coupled with a method for dealing with highly influential points, to be more appropriate for use in QRA.

Among the eight T2 trends in Table3 that show at least moderate evidence of an effect of REC (T2 *p* < 0.1) when radon is not adjusted, their T2 slopes differ by less than a factor of 2, ranging from 0.00026 μg/m^3^-year to 0.00047 μg/m^3^-year. Thus, quantitatively these eight REC estimates give roughly similar results, despite their differing definitions. However, with radon adjusted, none of the T2 trends are significant and six of the nine REC estimates show a negative trend (Table3).

There is also progressively less evidence of an effect of REC as the analysis is restricted more completely to underground as opposed to surface work (Tables5). This does not appear to be due entirely to loss of power in restricted analyses, since the trend slopes also become more attenuated as the analyses are restricted to underground work. Analyses restricted to workers who spent their entire work experience underground show no consistent effect of REC exposure (Table5). It is important to keep in mind that the surface-only workers had very low REC exposures and no radon exposure, as occurred with underground work.

Results in Tables5 are restricted to estimates of cumulative exposure lagged 15 years computed using different estimates of REC. Cumulative exposure lagged 15 years was chosen because this metric nearly always gave the strongest evidence of an association between REC exposure estimates and lung cancer mortality, regardless of the REC estimate used. This finding is consistent with the results reported by Silverman *et al*.^18^

In two earlier case-control studies of exposure to DEE and lung cancer mortality, both Steenland *et al*.^25^ and Garshick *et al*.^26^ concluded that cumulative exposure to DEE lagged five years gave the most consistent evidence of a relationship between DEE exposure and lung cancer. The Silverman *et al*.^3^ analysis, however, emphasized cumulative exposure lagged 15 years and did not report analyses that used lags of five years. Utilizing a lag of five years gives a much more attenuated slope compared with a lag of 15 years (Table2).

A QRA based on a meta-analysis of data from those three case-control studies combined results from Steenland *et al*.^25^ and Garshick *et al*.,^26^ which used cumulative REC exposure lagged five years, and from Silverman *et al*.,^3^ which used cumulative REC lagged 15 years, while using a lag of five years in calculations of excess risk (Vermeulen *et al*.^29^). As pointed out in Crump,^30^ this mixing of analyses conducted using different lags constitutes an “apples and oranges” comparison and is not appropriate. However, it should be noted that, contrary to the implication by Vermeulen *et al*.,^31^ Crump^30^ did not recommend using a five-year lag, only a common lag. A five-year lag was used for illustration by Crump^30^ because five-year and zero-year lags were the only common values of lag available from the three studies. The conceptual problem caused by combining exposure estimates based on different lags needs to be addressed in any QRA for DEE that utilizes data from different studies.

Moolgavkar *et al*.^21^ conducted parallel re-analyses of the DEMS cohort data using both parametric methods based on a multistage carcinogenesis model and the more conventional statistical approach (also used by the original DEMS investigators^17^) based on the Cox proportional hazards model. They reported that temporal factors in REC exposure were important in determining the association with lung cancer mortality, i.e., summary measures of exposure, such as cumulative REC, did not accurately capture the risk associated with exposure. They reported also that the relative risk of lung cancer associated with exposure to REC was strongly modified by age. Thus, temporal factors in both exposure and risk, neither of which was addressed in the Attfield *et al*.^17^ analyses, should be considered in QRA of exposure to DEE. Similarly, in the case-control data, when cases and controls were categorized into three groups by age, and an age × REC cross-product term was added to the model with cumulative exposure lagged 15 years based on DEMS_REC1 (Table2), the improvement in fit was highly significant, and the REC effect, although still highly significant, decreased with age (results not shown). Thus, if the DEMS data are used in QRA, the age effect on risk should be taken into account.

Moolgavkar *et al*.^21^ also reported that both in their parametric and proportional hazards analyses the REC-associated increased risk of lung cancer mortality was driven by the response in only one mine (mine A, the limestone mine). As noted above, our analysis did not confirm this finding, but indicated instead that no single mine was responsible for the REC-associated increased risk (Table6). However, there are a number of important differences between the two analyses, aside from the fact that one was based on the cohort data, and one on the case-control data. Moolgavkar *et al*. used the individual estimated REC exposures rather than summarizations and could not control for other covariables, including smoking and radon, because these data were not available. Thus, the reason for the difference in mine-specific results is not clear, but this issue also should be resolved before the DEMS data are used in any QRA.

In summary, we were able to replicate the findings reported by Silverman *et al*.^18^ when we used the same analytical methods. This gave us confidence that we were using the same basic data set as Silverman *et al*.^18^ We extended the analyses using alternative REC estimates developed by the DEMS investigators and obtained results similar to those reported by Silverman *et al*.^19^ We proceeded to apply six alternative REC metrics, five of which depended, as did the DEMS metrics, on extrapolations involving assumed relationships between CO and REC. A sixth REC metric, REC6, was used that did not involve any assumptions concerning the relationship between CO and REC, and was based on Adj_HP and ventilation rates for each of the mines. Of the several REC metrics, we view REC6 as having some superior qualities because it avoids using the highly uncertain assumptions concerning the relationship between CO and REC.

Most importantly, we used the radon concentration data for the DEMS cohort provided by the DEMS investigators. When adjustment was made for radon, a known human lung carcinogen, the effect of REC on the association with lung cancer mortality was confined to only the three DEMS REC estimates. Most notably, there was no evidence of an association with any of the six alternate REC estimates, including REC6. When T2 trend tests were conducted, based on the use of individual worker REC estimates, the results were less statistically significant and in many cases the trends were negative. Indeed, for miners who always worked underground, five of the six REC metrics exhibited negative trends.

The trend slopes in Table2 and other tables were derived from a conditional logistic regression analysis that assumed that the log odds of dying of lung cancer can be expressed as the sum of a factor that is independent of REC exposure and a factor equal to the product of REC exposure and the slope. The slope consequently is in inverse units of REC. If the probability of dying of lung cancer is approximated by the odds of dying of lung cancer, a slope of 0.00064 (μg/m^3^-y)^−1^ (Table2, cumulative exposure to DEMS_REC1 lagged 15 years, adjusted for “with radon” variables) represents an excess lung cancer probability of exp(0.00064) − 1 = 0.00064, times the baseline lung cancer risk, for individuals exposed to 1 μg/m^3^-year of REC, where the exposure might have been attained by REC exposure to 1 μg/m^3^ in one year or 0.1 μg/m^3^ per year for 10 years. Similarly, if exposure is to 132 μg/m^3^-y of REC (an amount that could be obtained, e.g., from a total of 20 years of constant outdoor work in the Bronx, where exposures average 6.6 μg/m^3^^18^), the excess lung cancer probability would be estimated as exp(0.00064 × 132) − 1 = 0.09, times the baseline probability. If the baseline probability of dying of lung cancer is 0.1 (rate for men unadjusted for smoking, see, e.g., Villeneuve and Mao^32^), the excess lung cancer probability would be 0.09 × 0.1 = 0.009, and the total probability of dying of lung cancer would be increased from 0.1 to 0.109 by the REC exposure. The reliability of such an estimate is obviously greatest for a population exposed to REC concentrations and duration of REC exposure similar to those encountered by participants in the DEMS case-control study. The foregoing material has been provided to illustrate how the results of various analyses can be interpreted and should not be viewed as endorsement of the particular numerical values used in the examples.

The results of our analyses indicate that the evidence for an association between exposure to DEE and lung cancer mortality in the DEMS case-control data set is less robust than indicated by the original reports. Our analysis also highlights a number of important issues that need to be addressed in any use of the DEMS data for QRA. This includes the quantitative differences in results based on different estimates of DEE exposures, whether an analysis should use personal exposure estimates versus a group average, and how to adjust the analysis for covariables other than DEE, including particularly radon.

## 8.EPILOGUE

The authors recognized from the beginning of their reanalysis efforts using the DEMS data that the endeavor and the results would be controversial. This controversy is grounded in four decades of research conducted, initially, to characterize the potential carcinogenic hazard of diesel exhaust exposure through the conduct of epidemiological studies, animal investigations, and mechanistic research and, in a second step, to establish the potency of diesel exhaust as a basis for conducting QRA. Although the controversy continued, regulations were issued in the United States and many other countries requiring the marketing of ultra-low sulfur fuel along with improved engine designs, including exhaust after-treatment systems to markedly reduce exhaust emissions of particulate matter and other pollutants. Many of these clean diesel engines are already in use on-road and in other applications in the United States and other countries. However, because of their durability, many traditional diesel engines manufactured before the introduction of the new technology diesel engines are still in use. Hence, the continued interest in having information on the potency of diesel exhaust from traditional engines for use in QRA.

The authors also recognized that some individuals and organizations might be concerned that the reanalysis of the DEMS data was being conducted with industry sponsorship and, they might charge, influence regarding the conduct of the reanalysis and the reporting of the findings. (Funding of the reanalysis reported herein was coordinated by the Truck and Engine Manufacturers Association [EMA] and the financial sponsors are listed in the Declaration of Interest.) Recognizing these concerns, the EMA selected a team of investigators based on their scientific credentials and allowed them total independence in developing a research strategy for conducting the reanalysis. It was agreed by the EMA and the investigators that the investigators would have exclusive responsibility for preparing the requests for access to the DEMS data, and related protocols for data reanalysis to be submitted to NIOSH and NCI to gain access to the data. The EMA did work with NIOSH and NCI to facilitate meetings between the original DEMS investigators and NIOSH personnel to gain access to the DEMS data. However, the EMA was not given access to the DEMS data. A representative of EMA was provided with earlier versions of this article and provided comments; however, the listed authors are responsible for the article in its entirety.

In developing the protocols for reanalysis of both the cohort and case-control data, the initial focus was on replicating the findings reported in the peer-reviewed literature by the original DEMS investigators. As an aside, recognizing the expertise of the DEMS investigators, it was fully anticipated that the results would be replicated. However, we viewed this step as important to validate the findings and to ensure that we were using the same basic data set for our extended analysis.

The strategy for conducting the extended analyses was clearly not an attempt to discredit the original findings. The goal was to determine if alternative analyses of equivalent or greater validity could be found that could be useful in developing a QRA for DEE using the DEMS data. The focus of the reanalysis of the case-control data reported in this article was on the effect of alternative DEE exposure assessments and adjustment for potential confounding variables, and in the reanalysis of the cohort data set, it was on using alternative exposure-response models.^21^

As is apparent from Section 7 of the article, the findings of the original DEMS investigators were, in fact, replicated. However, equally as important, we interpret our findings as having very significant implications for how analyses based on the DEMS data should be interpreted and used for QRA. To be specific, all of the analytical results on the DEMS cohort (which includes the case-control study), including (1) those reported by the original investigators in their papers,^17^–^19^ (2) the results reported on the reanalysis of the cohort data,^21^ and (3) the results in this article, should be considered in any attempt to develop estimates of cancer-causing potency for diesel exhaust.

It is hoped that the approach we have used to conduct this reanalysis, both the replication and the extended analyses, of the DEMS data set will serve as an example for similar efforts with other large complex data sets in investigating the potential health impacts of various potential risk factors. When complex data sets, such as DEMS, are acquired using public or private funding on issues of great importance to society, it is only prudent for multiple investigators, not just the original investigators, to analyze the data. Policy decisions based on the findings from these data sets, whether from original analyses or from reanalyses, can have large health and economic impacts. It is important to recognize that the most useful and revealing approach to analyzing large, complex data sets cannot always be anticipated when the initial protocol is developed. Moreover, different analysts have unique skills that can be used in conducting extended analyses. Finally, as demonstrated in this reanalysis effort, regardless of the funding source, investigators can be given full independence by and from the source of the funding so that it should not influence the scientific credibility of the results. It is our strongly held opinion that the scientific credibility of research should be judged based on the scientific quality of the work.

## Supporting Information

Disclaimer: Supplementary materials have been peer-reviewed but not copyedited.

**Table S1**. Number of miners and lung cancer deaths by worker location and mine type in the DEMS cohort. Two additional cases were removed in the case-control analysis **Table S2**. Table 2 of Stewart *et al*.^11^ summarizing the data available for constructing estimates of REC exposures at the eight mining facilities. **Figure S1**. Graphs of adjusted horsepower and mine ventilation rates by year. **Figure S2**. Comparison of imputed CO values of Vermeulen *et al*.^14^ with those of Crump and Vanlandingham^16^ showing longer tails of latter imputed values
