# Supplementary material for: Reanalysis of the DEMS Nested Case-Control Study of Lung Cancer and Diesel Exhaust: Suitability for Quantitative Risk Assessment
Source: Risk Anal. 2015 Apr 10;35(4):676–700. doi: 10.1111/risa.12371 (PMC4690516; doi:10.1111/risa.12371)

**SUPPLEMENTAL MATERIAL**

**Access to DEMS Data for Re-analysis**

The DEMS data set was originally assembled by NIOSH and NCI investigators under protocols approved by their institutional Review Boards governing human studies. This included consideration of related agreements with the National Center for Health Statistics (NCHS) designed to protect the confidentiality of vital data on individuals included in the DEMS study. NIOSH initially created means for scientists external to NIOSH to access DEMS data used in the cohort study, and NCI created means for outside scientists to access the DEMS data used to conduct the case-control study. As we began to evaluate those data sets, we determined it was necessary to link information from the data sets in order to conduct analyses in addition to those conducted by NCI (e.g., to apply different exposure measures than the one used in the case-control analysis). At that stage in our research, we were advised by the federal agencies that we were prohibited from linking the several data sets and were required to destroy all work based on any linkages we had already established. Alternatively, NCI, NIOSH and NCHS proposed that the DEMS data could be assembled and made available for on-site uses at a NCHS Research Data Center (RDC) in Hyattsville, MD. A precondition to any analyses that we conducted at the RDC was that our work had to be carried out under written protocols pre-approved by RDC officials. A further condition was that upon completing our work at the RDC, we would have to leave behind the results of analyses for review by RDC personnel to ensure that all of our analyses had been conducted in accord with the pre-approved protocols and that all of our results satisfied the confidentiality rules of NCHS. All of the analyses conducted at the RDC were performed by Crump and Van Landingham. The other authors of this paper did not have access to the DEMS data at the RDC, and participated in the preparation of this paper by review and interpretation of results approved for release by the RDC.

**Further Description of Alternative Estimates of REC**

We did not use the 1998-2001 estimates of REC exposures developed by DEMS^(11-12)^. Instead, we developed independent estimates using the 1998-2001 REC data and mirroring the DEMS approach^(16)^.

Crump and Van Landingham^(16)^ made independent imputations for non-detect CO samples using an alternative (and what was believe to be a superior) statistical approach. Whereas Vermeulen et al.^(14)^ fit separate log-normal models to the MIDAS and DEMS CO samples, we fit mine-specific models to the MIDAS data, and a separate model to the DEMS data. Also, since statistical tests firmly rejected the Vermeulen et al.^(14)^ assumption that the sets of CO samples were log-normally distributed (p = 6 x 10^-23^ for MIDAS data and p = 7 x 10^-8^ for DEMS data), we fit three statistical distributions (Log-normal, Weibull and Gamma) to each of the nine CO data sets we analyzed (eight mine-specific MIDAS and DEMS) and in each case used the distribution that fit the data the best. (See Crump and Van Landingham^(16)^ for a more complete description of these methods.) Figure 1 indicates that our method for assigning values to non-detects let to substantially longer-tailed distributions than those obtained by Vermeulen et al.

By regressing area samples of REC collected in the 1998-2001 survey on area CO samples, DEMS obtained a relationship of REC ~ CO^0.58^ ^(18)^. Nevertheless, the exposure estimates used in the case-control analysis^(18)^ were based on an assumed linear relationship (REC ~ CO^1.0^). However, in the Crump and Van Landingham^(16)^ regression analysis of 1998-2001 CO and REC data, we could not reproduce the exponent of 0.58. Instead a best estimate relationship of REC ~ CO^0.30^ was obtained. Herein we investigate the effect of these differing exponents by considering two values for the exponent in the REC – CO relationship (1.0 and 0.3).

Essentially like the DEMS REC estimates, we made year-, mine-, department-, and job-specific REC estimates by multiplying the 1998-2001 mine-, department- and job- specific REC estimates by

$$\left( \frac{{CO}_{y}}{{CO}_{ref}} \right)^{\beta},$$

where ${CO}_{y}$ is the is the estimated mine-specific value for CO in year y, ${CO}_{ref}$ is the mine-specific value of CO obtained from the DEMS survey in 1998-2001, and $\beta$ is the coefficient in the REC – CO relationship (either 1.0 or 0.3). See Crump and Van Landingham^(16)^ for additional information.

We also developed estimates of yearly CO levels defined as three-year averages of CO measurements, where the intervals were expanded to include adjacent years when there were fewer than 10 measurements in a given three-year period. These estimates were used as a replacement for the CO estimates obtained from the regression model (REC5). For year $y$ prior to 1975 (for which there were no CO measurements) we used

$${{CO}_{ym}=\left( \frac{\frac{{HP}_{ym}}{{CPM}_{ym}}}{\frac{{avgHP}_{75-79m}}{{avgCPM}_{75-79m}}} \right)}^{\beta_{1m}}{CO}_{75-79m},$$

where ${CO}_{ym}$ is the CO estimate for mine $m$ and year $y$, ${avgHP}_{75-79m}$ and ${avgCPM}_{75-79m}$ are, respectively, the average horsepower and average mine ventilation rate for 1975-1979 and mine $m$, $\beta_{1m}$ is the mine-specific coefficient from eq. 2 of Crump and Van Landingham^(16)^ (the CO regression model) estimated using all of the CO data, and ${CO}_{75-79m}$ is the average of the CO measurements in mine $m$ during 1975 to 1979. In addition, in several of the mines, the CO samples collected by DEMS in 1998-2001 are all systematically higher that the MIDAS samples for the same mine in 1995-1999 (see, e.g., Vermeulen et al.^(14)^ Table 1.), which we believe are unlikely to reflect real differences in CO, but are likely due to a difference in sampling or CO analysis techniques in the different surveys. To account for these differences, the sample averages from the 1998-2001 DEMS survey were multiplied by the appropriate factors based on the regression coefficients for “survey” obtained from the regression analysis, in order to make the DEMS data more comparable to the MIDAS data. These CO estimates were used in the same way as those obtained from the CO regression models to estimate REC exposures.

**DEMS POPULATION**

The DEMS population studied is summarized in Table S-I as an aid to the reader.

Table S-I. Number of miners and lung cancer deaths by worker location and mine type in the DEMS cohort. Two additional cases were removed in the case-control analysis

|  |  | |  | **Ever-Underground Workers** | | | |  |  | |
| --- | --- | --- | --- | --- | --- | --- | --- | --- | --- | --- |
|  | **Surface-Only**  **Workers** | | **Underground-**  **Only Workers** | | | **Surface and**  **Underground**  **Workers** | | **Complete**  **Cohort** | | |
|  |  |  |  |  |  |  |  |  |  |  |
| **Mine Type** | **Miners** | **Deaths** | **Miners** | | **Deaths** | **Miners** | **Deaths** | **Miners** | | **Deaths** |
| Limestone | 730 | 15 | 123 | | 12 | 823 | 10 | 1676 | | 37 |
| Potash | 1293 | 38 | 1951 | | 46 | 1327 | 18 | 4571 | | 102 |
| Salt | 50 | <5 | 208 | | 9 | 289 | <5 | 547 | | <19 |
| Trona | 1935 | 23 | 1798 | | 15 | 1788 | 11 | 5521 | | 49 |
| Entire cohort | 4008 | <81 | 4080 | | 82 | 4227 | <44 | 12315 | | <207 |

Table S-II. Table 2 of Stewart et al.^(11)^ summarizing the data available for constructing estimates of REC exposures at the eight mining facilities.

Figure S-1. Graphs of adjusted horsepower and mine ventilation rates by year


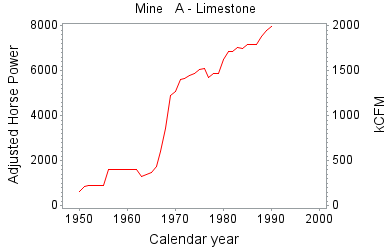

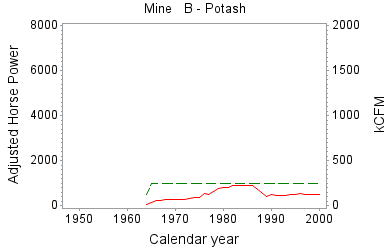


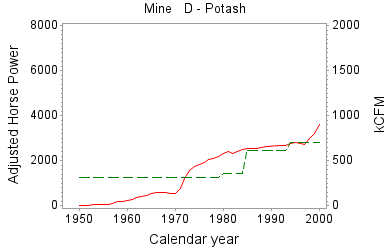

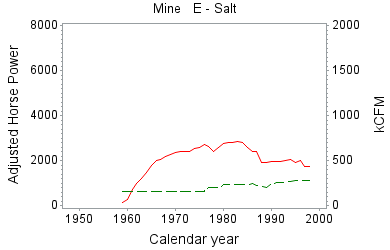


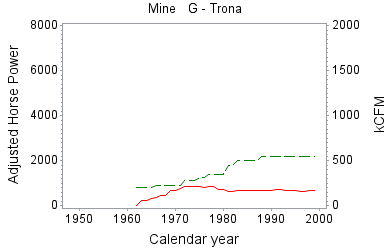

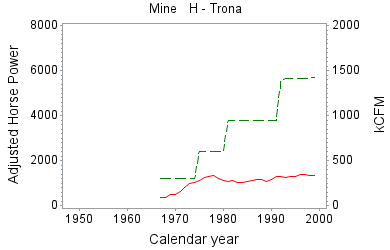


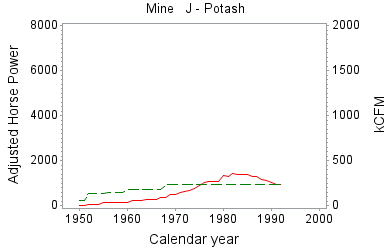

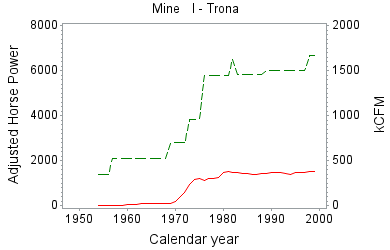


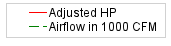


Figure S-2. Comparison of imputed CO values of Vermeulen et al.^(14)^ with those of Crump and Vanlandingham^(16)^ showing longer tails of latter imputed values


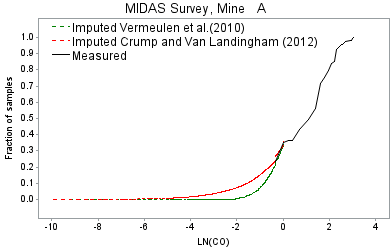

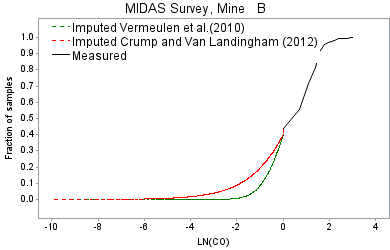

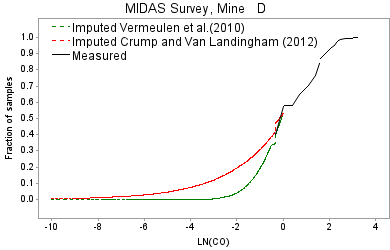

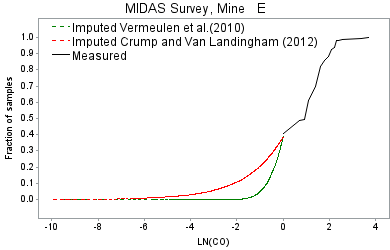

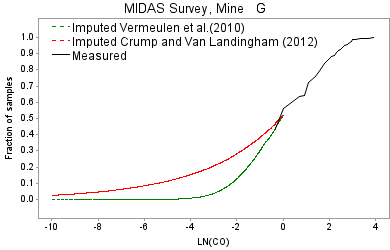

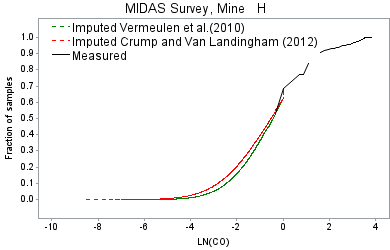

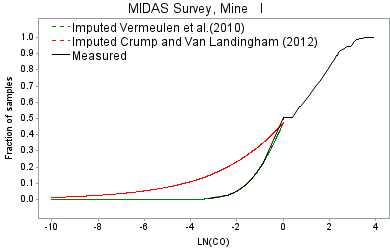

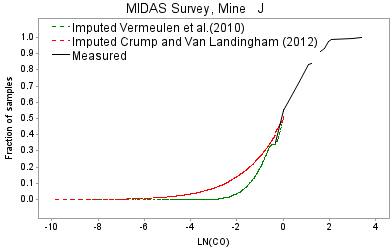

Supplement: Supplementary file 1 [file risa0035-0676-sd1.docx]
